# Combining Bayesian optimization and automation to simultaneously optimize reaction conditions and routes†

**DOI:** 10.1039/d3sc05607d

**Published:** 2024-04-29

**Authors:** Oliver Schilter, Daniel Pacheco Gutierrez, Linnea M. Folkmann, Alessandro Castrogiovanni, Alberto García-Durán, Federico Zipoli, Loïc M. Roch, Teodoro Laino

**Affiliations:** a IBM Research Europe Säumerstrasse 4 8803 Rüschlikon Switzerland oli@zurich.ibm.com; b National Center for Competence in Research-Catalysis (NCCR-Catalysis) Switzerland; c Atinary Technologies Route de la Corniche 4 1066 Epalinges Switzerland

## Abstract

Reaching optimal reaction conditions is crucial to achieve high yields, minimal by-products, and environmentally sustainable chemical reactions. With the recent rise of artificial intelligence, there has been a shift from traditional Edisonian trial-and-error optimization to data-driven and automated approaches, which offer significant advantages. Here, we showcase the capabilities of an integrated platform; we conducted simultaneous optimizations of four different terminal alkynes and two reaction routes using an automation platform combined with a Bayesian optimization platform. Remarkably, we achieved a conversion rate of over 80% for all four substrates in 23 experiments, covering *ca.* 0.2% of the combinatorial space. Further analysis allowed us to identify the influence of different reaction parameters on the reaction outcomes, demonstrating the potential for expedited reaction condition optimization and the prospect of more efficient chemical processes in the future.

## Abstract 2

Combining a cloud-based Bayesian optimization platform with a robotic synthesis platform accelerated the discovery of high conversion iodination of terminal alkyne reactions in a large search space of over 12 000 possible reactions in 23 experiments.

## Graphical abstract

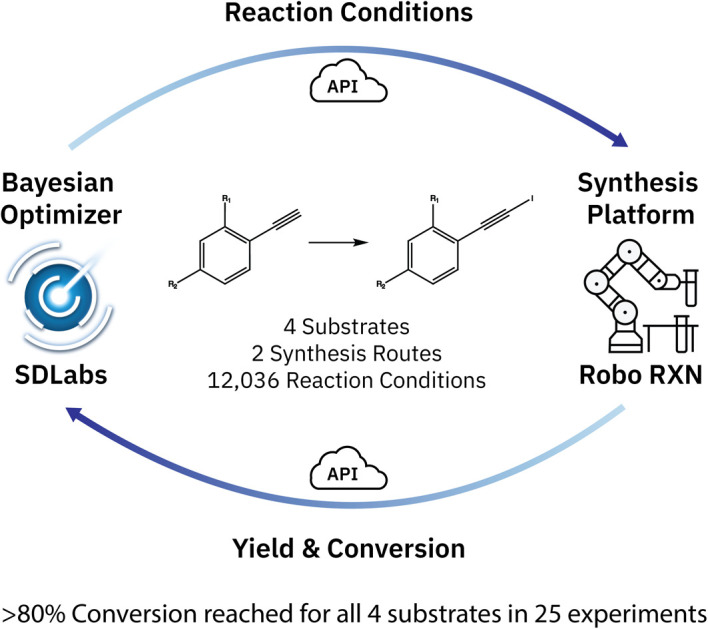

## 1 Introduction

Accelerating the research and development (R&D) of new chemical reactions plays a central role in improving various industries and addressing global challenges; it is a driving force behind technological innovation, sustainability, and societal benefits.^1^ Not only do new chemical reactions expand our understanding of fundamental chemical principles and mechanisms, but they are also at the heart of many industries, including pharmaceuticals, materials, agriculture, energy, and electronics.^2–9^

Discovering, developing, and optimizing chemical reactions lead to the creation of new, more efficient, and more sustainable products, technologies, and processes, enabling the possibility of revolutionizing these industries.^10–15^ However, finding the most optimal reaction path and conditions is a complex problem. Traditionally, these processes relied on trial-and-error approaches, including techniques such as one factor at a time (OFAT^16,17^) or design of experiments (DoE^18^) and on the expertise of researchers and scientists.

As R&D undergoes continuous digitization and is fueled by the abundance of untapped data and impressive advancements in artificial intelligence (AI), there arises a growing imperative to adopt a data-driven methodology.^19–22^ This adoption will empower scientists and researchers to utilize AI and to learn from past experiments, recognize patterns in the data, and ultimately suggest the next best experiments expediting R&D.^23^

One prominent trend in this data-driven approach involves the rising popularity of Bayesian optimization^24^ (BO) and reinforcement learning in recent years.^25–32^ Notably, deep reinforcement learning techniques were used along with domain knowledge of chemistry to improve the yield of four reactions carried out in microdroplet reactors.^28^ Similarly, Taylor *et al.*^33^ used a flow-based reactor to optimize the Suzuki–Miyaura coupling (3 continuous variables and one categorical) and C–H activation (5 continuous variables) *via* multi-task BO in 5–22 experiments, thereby reducing R&D costs over conventional optimization techniques. In a different study, BO techniques were used to improve the yield of the Heck cyclization-deprotection^34^ in a flow system based on an automated continuous flow platform parametrized by 4 input control variables such as the residence time, equivalents and temperature. The authors were able to achieve a yield of 81% in just 14 h (13 total experiments) and discovered a favorable competing pathway. Such findings highlight the versatility and potency of BO in diverse R&D scenarios.

One key challenge of incorporating BO methods in R&D is that these methods are typically considered as black boxes with limited explainability and interpretability,^35^ hindering their widespread adoption. Additionally, when the search space is large, researchers face difficulties in visualizing and understanding the way that the parameters influence the objectives. Though non-linear dimensionality reduction techniques such as t-SNE (t-distributed stochastic neighbor embedding)^36^ or UMAP (uniform manifold approximation and projection)^37^ have been used in chemistry applications such as anomaly detection, process control,^38^ and biomolecule molecular dynamics simulations,^39^ their application in BO models is limited. For example, Shields *et al.*^9^ used t-SNE to cluster BO samples in optimization campaigns, but the approach did not address the objective space or surrogate model predictions, leaving the relationship between inputs and objectives unexplored. Moreover, chemistry applications involve categorical variables and constraints which can pose challenges to conventional dimensionality reduction techniques. Successful application of these techniques to BO methods can shed light on the mechanisms by which BO methods can efficiently optimize problems.

To further enhance the speed and efficiency of R&D, conducting suggested experiments using automated robotic hardware and automatically feeding their results back to the AI platform can be highly beneficial. This integration of AI and automation is commonly referred to as a self-driving lab.^40,41^ These self-driving labs mark a revolutionary advancement in R&D, redefining our perception of time efficiency and of what is possible. Self-driving labs are like time machines, presenting the chemical and advanced materials sector with an opportunity to reshape their R&D processes, accelerating their pipelines to address challenges across the board. Recently, national and international efforts have led to the development of a hub towards democratizing these smart automated and data-driven approaches.^42–46^

An area where these self-driving labs represent a transformative leap is reaction optimization, where researchers systematically explore reaction conditions to identify the optimal set of experimental conditions and parameters for a desired chemical transformation.^11,26,27,47,48^

In this study, we integrated the machine learning (ML) engine from Atinary Technologies (SDLabs) and the robotic platform from IBM Research (RoboRXN) to demonstrate the autonomous optimization of the iodination of terminal alkynes within a complex search space with categorical variables and several constraints.

**Fig. 1:**
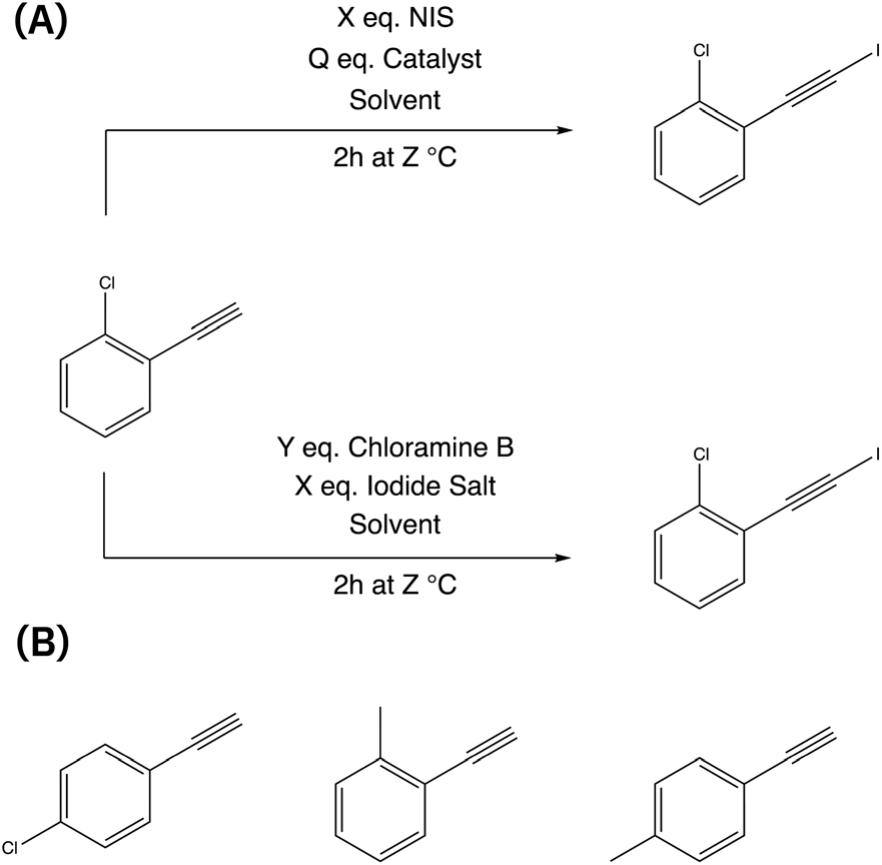
Iodination of 1-chloro-2-ethynylbenzene using two different routes (A) and the alternative three different aromatic alkynes (B) that are simultaneously optimized.

This reaction was chosen due to its significance in enabling the synthesis of complex organic molecules by introducing an iodide group while preserving the alkyl group. The resulting compounds can undergo various subsequent transformations, such as nucleophilic additions,^49^ cross-coupling reactions,^50^ and cycloaddition,^51^ allowing for further functionalization of iodoalkynes.^52,53^ There are multiple chemical routes available for achieving iodoalkynes (see Fig. 1). In this study, we focused on two different routes simultaneously to determine the most effective reaction conditions: (i) the first route utilizes *N*-iodosuccinimide (NIS) as the iodinating agent,^54^ while (ii) the second route involves chloramine-B as the oxidant and iodine salts as the halogen source.^55^

We optimized the reaction using three distinct Bayesian optimizers. Impressively, two out of the three optimizers achieved over 80% conversion for all four reactants within just 23 reactions. To gain insights into the underlying reasoning of BO algorithms, we employed various visualization techniques.

In particular, we introduced a contour plot based on t-SNE dimensionality reduction, specifically designed to visualize categorical spaces. This tool allowed us to enhance the interpretability of BO methods, shedding light on their ‘black box’ nature and providing a more transparent, comprehensible approach to self-driving labs.

## 2 Methods

Here, we provide details on the execution of the reaction, on the integration of the platforms and on the AI algorithms employed, as well as outlining the closed-loop optimization workflow.

### 2.1 Reaction execution

The reaction conditions were assessed with two objectives: achieving a high conversion of the alkynes and maximizing product yield. The reaction parameters included the choice of solvent, the concentration of reagents (catalyst, iodine source, iodinating agent, and solvent), and reaction temperature (see Table 1). Throughout the experiments, a standardized procedure was followed, with a fixed reaction time of 2 h and a total reaction volume of 20 ml for all runs. In order to explore a broader range of starting materials, four different aromatic alkynes were simultaneously screened (see Fig. 1).

**Table 1:** All reaction parameters selected by the optimizer

Parameter	
Alkyne group	2-Ethynyltoluene
	4-Ethynyltoluene
	1-Chloro-2-ethynylbenzene
	1-Chloro-4-ethynylbenzene
Chloramine [eq.]	0, 1, 1.5, 2
Solvent	MeOH, CH_3_CN/H_2_O, THF, DMF, DMSO, DCM, EtOAc, 1,4-dioxane, MTBE, DCE
Iodine source	KI, NaI, TBAI, NH_4_I, NIS
Iodine source [eq.]	0, 1, 1.5, 2
Catalyst	PSTA, AcOH, none
Catalyst [eq.]	0, 0.1, 1
Temperature [°C]	25, 35, 45, 55, 65, 75, 85, 95

Solutions of the internal standard and starting alkynes were prepared in acetonitrile at a concentration of 1 M. 100 ml stainless steel reactors were preloaded with the required iodide salts and iodinating reagents. Subsequently, the robotic platform added the solvent, followed by 1 ml of a 1 M solution of the alkynes. If applicable, a catalyst was added by the robotic platform. The quantity of solvent was carefully adjusted to ensure a consistent reactor concentration of the alkyne, maintaining a total reaction volume of 20 ml. The mixture was then heated to the desired temperature and stirred for 2 hours under a nitrogen atmosphere. After 2 h, the mixture was cooled to 25 °C, and the reaction was quenched by adding 1 ml of a saturated thiosulfate solution (in acetonitrile) followed by 1 ml of an internal standard (1 M acetophenone in acetonitrile). A 0.3 ml sample of the reaction mixture was automatically diluted 50x with acetonitrile and filtered. From this diluted solution, a 5.0 μl sample was injected by the robotic platform into an HPLC/diode array detector (DAD) setup for analysis. A more detailed example synthesis procedure can be found in Section A.5 of the ESI.†

### 2.2 Reaction parameter space

A multitude of potential combinations of reaction conditions can be generated, resulting in a vast grid of potential candidate conditions. To handle this efficiently, continuous variables such as temperature were discretized into intervals (*e.g.*, 10 °C intervals), and equivalent reagents of 1 eq., 1.5 eq., and 2 eq. were considered. This approach allowed managing the size of the grid. The optimized parameters are listed as follows: alkyne group (4 options), iodine source (5 options), iodine source amount (4 options), solvent (11 options), catalyst (3 options), catalyst amount (4 options), chloramine amount (4 options), and temperature (8 options) and can be found in Table 1. It is important to point out that certain constraints were imposed on the grid of possible reaction conditions. For example, the maximum allowed reaction temperature at each grid point was set to the boiling point of the associated solvent to prevent evaporation from affecting the reaction. Additionally, reagents involved in the reaction, such as chloramine salts, were required to be used in at least stoichiometric amounts (over 1 eq.). Combinations of reactants that were not involved in the reaction mechanism were excluded to avoid interactions that would not contribute to the desired outcomes. For instance, NIS and chloramine salts or chloramine salts with the acid catalyst typically used alongside NIS were not combined. With these restrictions, we ended up with a grid size of 12 036 combinations, which makes it impractical to extensively evaluate all the possible combinations. The full grid can be found in the ESI.†

### 2.3 Yield and conversion measurements

To assess the yield and conversion of the formed products and iodoalkynes, the DAD at 254 nm of the HPLC setup was used. This detector is suitable since all starting materials and their corresponding products are UV-detectable. An internal standard, acetophenone solution (1 M in acetonitrile), was added (1 ml) into the reactor vessel after the reaction was completed and quenched. This internal standard also allowed the detection of errors during the injection of a sample. To correlate the peak area with concentration in the reaction mixture, calibration curves were obtained. These curves and synthesis procedure for reference materials can be found in the ESI (see Sections A.5 and A.6†) and allowed the determination of yield and conversion. Since the amount of starting material and the amount of internal standard (both 1 ml of 1 M solutions) are equivalent, the internal standard concentration can be used as a proxy during the yield and conversion calculations, which are defined as follows:1
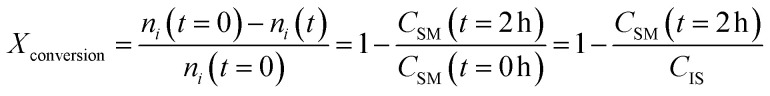
where *C*_SM_ is the concentration of the starting material. This corresponds to the fraction of consumed alkyne at *t* = 2 h divided by the originally added concentration of alkyne at *t* = 0 which is equal to the concentration of the internal standard *C*_IS_. The yield is defined as follows: w2
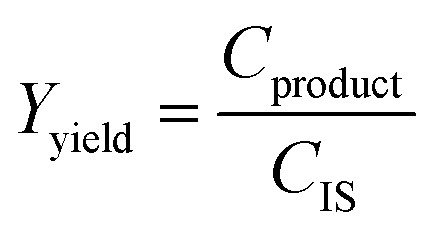
here *C*_product_ is the product concentration normalized by the internal standard concentration *C*_IS_. Due to a slight misalignment of the calibration curve intercept with the zero point on the *y*-axis, it is possible that certain reactants and their corresponding products exhibit a higher yield or conversion than theoretically expected. This discrepancy is likely attributed to the accumulation of experimental errors stemming from factors such as the preparation of handmade mixtures, the variability in injection reproducibility, and concentration fluctuations in the stock solutions due to evaporation, among others.

### 2.4 Platform integration and closed-loop optimization

**Fig. 2:**
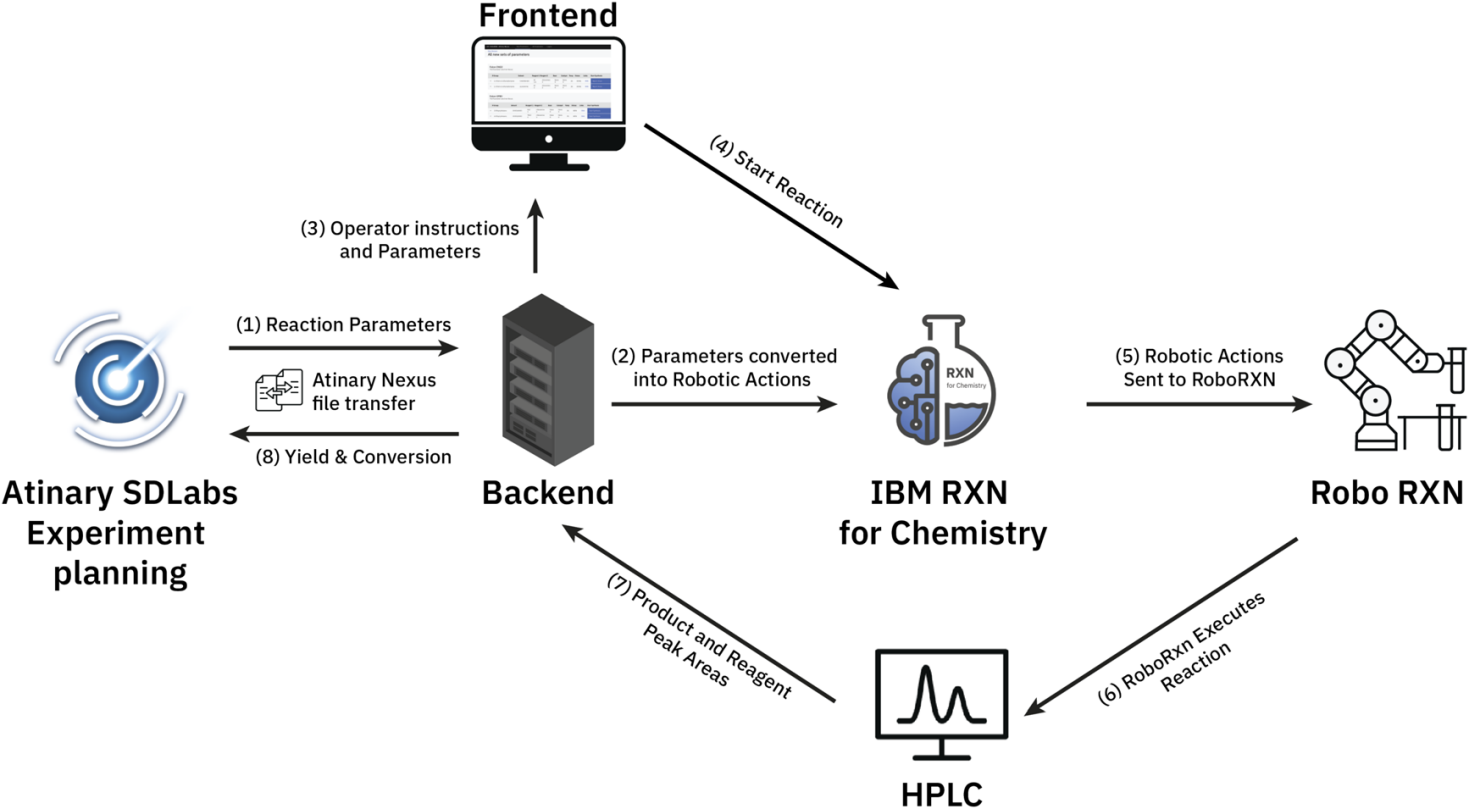
The workflow between the two platforms: (1) first, the backend application queries the Nexus platform to check if there are new reaction parameters to be processed. (2) If new parameters are found, the backend application converts them into a set of robotic actions that can be executed on the IBM RoboRXN platform. (3) Additionally, the reaction parameters are also translated into mass and volume units so that the operator can load the required reactants into the robotic platform. (4) Once the reactants are loaded, the operator can start the reaction using the web application. (5/6) This triggers the execution of the robotic actions on the RoboRXN platform. (7) After the reaction is completed, the operator manually checks the HPLC DAD-chromatogram to verify the results and enters the relevant peaks and their corresponding areas *via* the web application. (8) Finally, the backend application automatically calculates the yield and conversion based on the entered peak areas and returns them back to Atinary SDLabs.

We established a seamless workflow between the two cloud systems: IBM's RoboRXN‡‡https://rxn.res.ibm.com. robotic synthesis platform and the ML-driven Atinary SDLabs§§https://atinary.com. platform. We did so by creating a backend application that facilitates communication between the two platforms using a JSON file containing the reaction parameters, which is accomplished *via* Atinary Nexus, Atinary's proprietary file exchange platform (see Fig. 2).

When new reaction parameters become available, the backend application queries the Nexus platform to process them into a synthesis procedure. This synthesized procedure is then automatically executed on the RoboRXN platform.

**Fig. 3:**
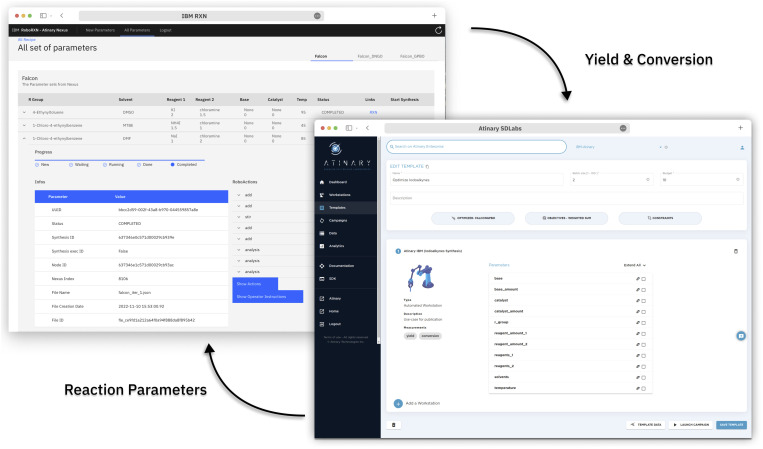
The graphical user interface of the Atinary SDLabs and the frontend application. SDLabs allows the user to configure the optimization campaign, while the frontend facilitates the execution of the reaction and reporting of the measured yields and conversions.

The operator can access the reaction status and relevant information through a frontend web application (see Fig. 3), which provides instructions on the amount of chemicals to load into the robot and enables the operator to return the result of the HPLC DAD-chromatogram. Once the peak area of the chromatogram is returned, the backend application calculates the conversion and yield based on before-run calibrations and returns these values to Atinary SDLabs. Overall, this workflow enables a seamless communication between the two platforms and facilitates the automation and optimization of chemical synthesis.

Three algorithms were used to optimize the experimental and process conditions of the iodination of terminal alkynes. These included three flavors of Atinary Falcon (Falcon Light, Falcon DNGO (deep neural network global optimization) and Falcon GPBO (Gaussian process Bayesian optimization)), a suite of general-purpose algorithms, which can solve optimization problems that include continuous, discrete and/or categorical variables with or without physicochemical descriptors, as well as batch-constrained optimization. These algorithms are defined in terms of their surrogate model and their acquisition function. The surrogate model is a probabilistic method that predicts the objective values and uncertainty from the input parameters. The acquisition function is a policy that will select the next points to evaluate given the surrogate model: it balances the trade-off between exploration (sampling from areas where the uncertainty is high) and exploitation (sampling from areas where the predicted value is optimal). The yield and conversion were jointly optimized for each of the alkyne groups. The stopping (convergence) criterion for each alkyne group was set to be surpassing an 80% conversion. In order to jointly optimize the yield and the conversion, a weighted-sum scalarization was implemented providing a weight of 0.9 to the normalized yield and 0.1 to the normalized conversion. This weighting scheme was designed to prioritize yield significantly more than conversion during the optimization process. However, conversion was still accorded a measure of importance to favor reactions showcasing the reactivity of the starting material over reaction conditions that experience no conversion at all. The result of this weighted-sum function is from now on referred to as merit. The obtained merit from the multi-objective function was chosen as the maximization target for all the optimizers.

Given that RoboRXN has a total of 6 reactors available anytime, we opted to utilize parallelization. Instead of sending a single set of parameters, we opted to send a set of two reaction parameters simultaneously, which enabled us to run two reactions in parallel for each of the three algorithms.

## 3 Results and discussion

For the three optimization campaigns, an initial set of 11 sampled grid points (out of the 12 036 possible combinations) were picked as starting conditions. After the initial sampling, the conditions used in the subsequent loops were recommended by the ML algorithms, in batches of 2 experiments in each round, until the stopping condition (conversion >80%) is met for all the alkyne groups. The initial sampling approach maximized sample diversity by employing a greedy heuristic, where the grid points were sequentially selected based on how many new unseen categorical options they explored. This allowed us to cover all the solvents, alkyne groups, iodine sources, and catalysts in 11 experiments. All the optimizers shared the same initial 11 points, but continued the optimization independently. The stopping condition for each alkyne was reaching a conversion of 80%, which would lead to excluding this substrate from the search space.

**Fig. 4:**
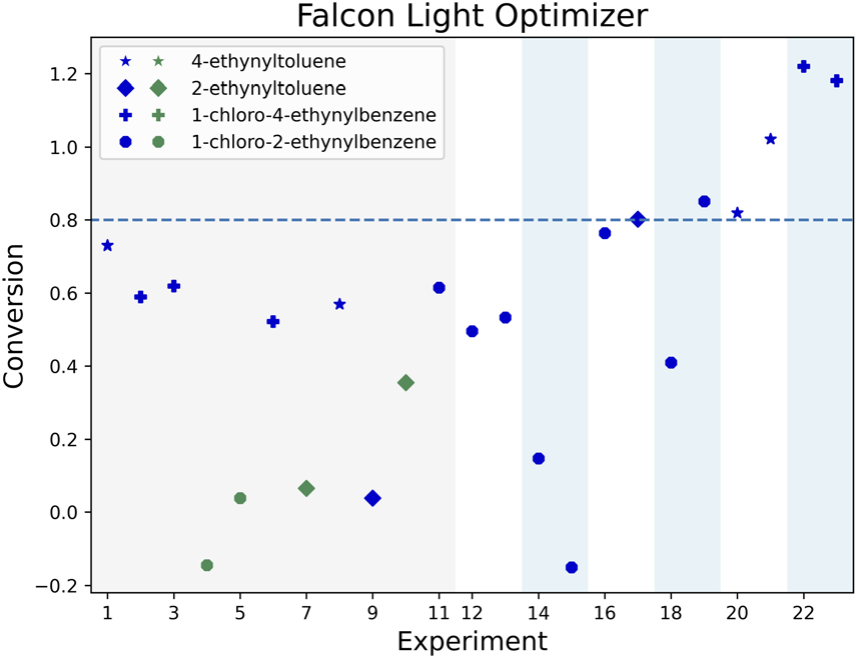
Conversion progress chart for the Falcon Light campaign. The gray area corresponds to the initial 11 reactions. Each pair of parameters is marked with a change in background color, the shape of the dots indicates the alkyne used, while the color indicates if the NIS (green) or chloramine (blue) route was used. If an alkyne is converted over 80% (dotted line), the alkyne is no longer further optimized. The optimization campaign ends when all four substrates reach 80% conversion.

Fig. 4 illustrates the joint optimization of the conversion for the four alkynes in 23 experiments for the Falcon Light optimizer. The marker shapes and colors correspond to the alkyne group and the reaction pathway used (either NIS or chloramine). The figure shows a fairly low distribution of conversion for all alkyne groups in the initial 15 experiments (3 iterations), and then shows that the convergence criterion is reached for 2-ethynyltoluene at experiment 17, followed by 1-chloro-2-ethynylbenzene at experiment 19, 4-ethynyltoluene at experiment 20 and 1-chloro-4-ethynylbenzene at experiment 22. Regarding the reaction pathways, 4 out of the initial 11 points featured NIS (green), and the remaining used chloramine. After this initial set, only the chloramine route is exploited by this algorithm.

Even though most of the experiments were performed with 1-chloro-2-ethynylbenzene (circle in the Fig. 4), these optimality conditions were leveraged to optimize the other alkynes. In fact, only two experiments used 4-ethynyltoluene before converging and only three experiments used 1-chloro-4-ethynylbenzene and 2-ethynyltoluene before they converged, compared to 10 experiments required to optimize 1-chloro-2-ethynylbenzene. Similar results can be seen for the other two optimizers (see Fig. A.1 and A.2 in the ESI†). The following sections compare the optimal conditions across alkyne groups to further illustrate how their information was transferred by the optimizers.

**Fig. 5:**
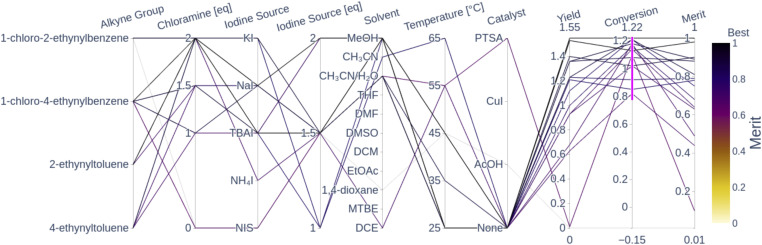
Parallel coordinates of the 16 converged experiments (conversion above 80%) from the three optimization campaigns. For the sake of clarity, the remaining experimental conditions that lead to a conversion below 80% are not displayed. Each line corresponds to an experiment and the columns correspond to the different variables (parameters, objectives and weighted-sum merit). The highlighted traces correspond to the experiments with a conversion surpassing 80%. The merit corresponds to a normalized weighted sum; the higher the weighted sum the better the performance. The traces are color-coded by the merit (*i.e.* darker traces indicate better performance than lighter ones). The plot with all 49 observations is shown in Fig. A.24 in the ESI.†

Fig. 5 shows a parallel coordinates chart illustrating the global optimality regions from all the seen experiments in terms of their measured conversion, yield and computed weighted-sum merit to be maximized (0.9 yield + 0.1 conversion). Every path represents an experiment; the left columns display the parameters while the columns on the right show the measurements (objectives). High values for merit correspond to good performance and low values correspond to poor performance as per the color-coding.

The parallel coordinates plot shows that there are multiple optimal conditions for the four alkyne groups, all using chloramine, and only one using NIS. The chloramine paths (chloramine amount above 1 eq.) feature at least 1 eq. of any reagent, 1 excluding NIS, polar solvents such as MeOH, CH_3_CN and CH_3_CN/H_2_O, temperatures below 65 °C, and no catalyst. These results correspond to the optimality values reported by Liu *et al.*,^55^ where they report the highest yield at room temperature in CH_3_CN. The only NIS route uses CH_3_CN/H_2_O as a solvent, a PTSA catalyst, 1.5 eq. of NIS and a temperature of 55 °C, which resulted in a conversion above 80%, but still resulted in a near-zero yield and a poor merit value. A plausible explanation for the low yield observed from this reaction condition is the fact that PTSA, as a relatively strong acid, can cause electrophilic hydration of the alkyne group to the corresponding ketone. This reaction is reported in the literature by Liu *et al.*,^56^ which explains the high conversion with corresponding low yield. The fact that uncatalyzed NIS reactions showed a low yield and conversion corresponds to the observation by Yao *et al.*,^54^ where they showed that the chemoselectivity and regioselectivity are driven by the presence of an acid catalyst. The absence of the catalyst can lead to the formation of a diiodovinyl substrate instead of the desired product. The literature-reported optimal conditions for the NIS route feature temperatures above 70 °C and the AcOH catalyst, which was not visited in any of the experiments. All of the seen NIS experiments resulted in poor performance, which seemed to direct the optimizers to focus on the more robust chloramine route. The following sections visualize the performance of the Bayesian optimizer as a function of the parameter space and the seen experiments.

**Fig. 6:**
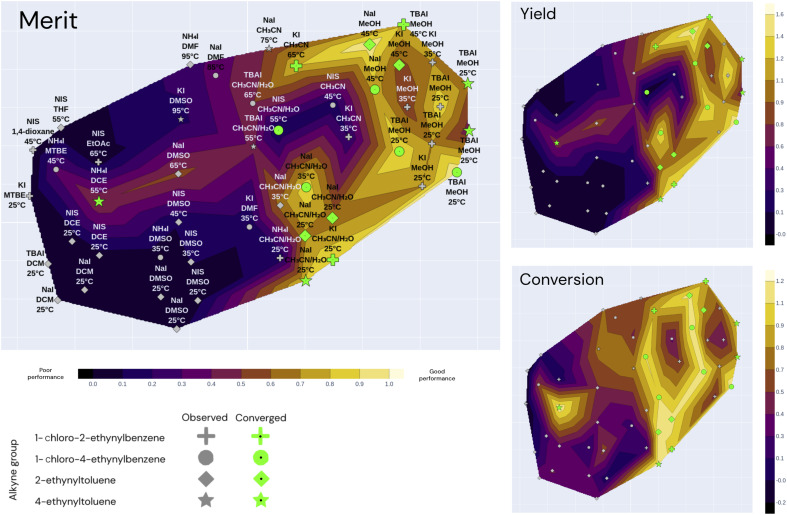
Annotated t-SNE plot with the true merit, conversion, and yield using all 49 observations coming from the 3 independent optimization campaigns (Falcon Light, Falcon GPBO and Falcon DNGO), each having the same 11 starting conditions and 12, 12, and 14 additional experiments, respectively, adding up to a total of 49. The *x* − *y* coordinates correspond to the t-SNE components, markers correspond to past experiments, and shapes correspond to alkyne groups. The marker annotations correspond to the iodine source, solvent and temperature associated with the experiments (other parameters such as reagent amounts and catalyst are not shown). Nearby markers correspond to experiments with similar parameter values, and the alkyne group value was not included in the dimensionality reduction. The color code of the contour map corresponds to the true objectives (merit, yield and conversion): lighter colors correspond to optimal regions and the dark regions correspond to suboptimal regions. Green markers correspond to converged experiments (>80% conversion).

**Fig. 7:**
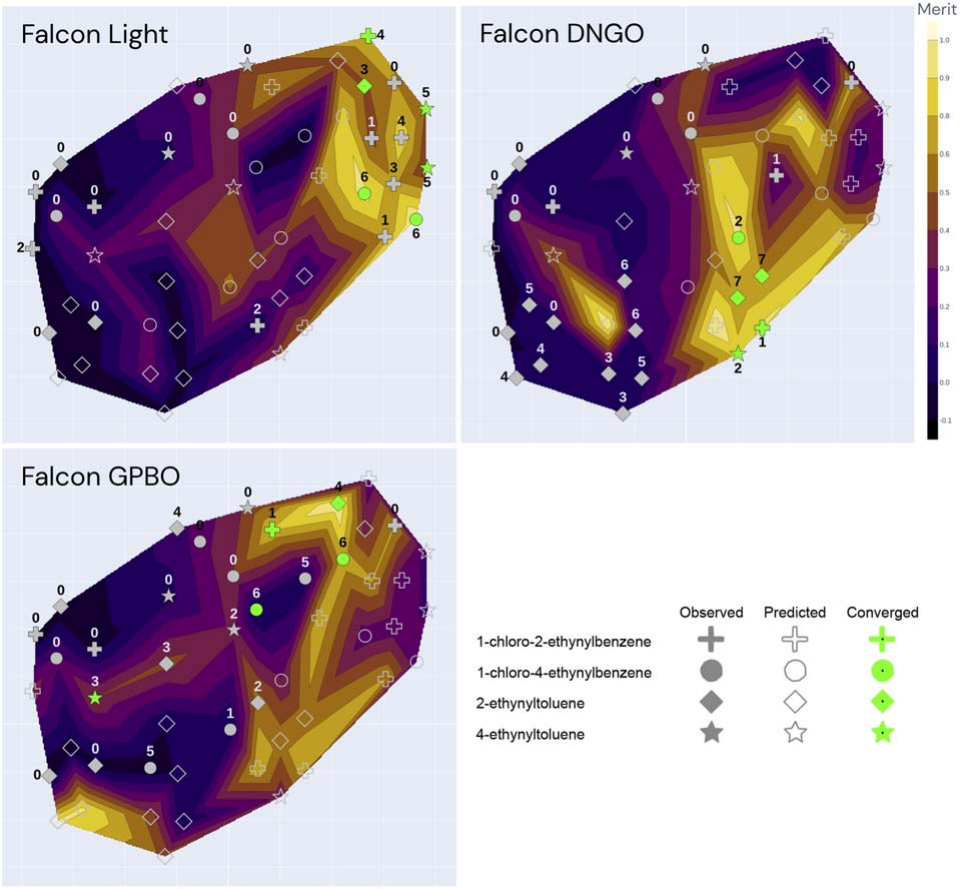
t-SNE plot for the three optimization campaigns (Falcon Light, DNGO and GPBO). The marker annotations correspond to the iteration number of the specific campaign. The color-code corresponds to the predicted surrogate model using only the labelled (closed) markers as training data. A fully annotated version of these plots is shown in the ESI.†

A contour plot was generated to visualize the objective values and surrogate model predictions of the Bayesian methods. This contour plot is the result of reducing the parameter space to 2 dimensions and setting the elevation to be the objective values at the given iteration. Fig. 6 shows an example plot with the seen objective values, and Fig. 7 illustrates the same plot showing the surrogate-model predictions instead, which allows a visual qualification of the models' predictive performance when compared with the ground truth. Each experiment is shown as a marker in the map: similar experiments appear close to each other in the contour plot. To reduce the high-dimensional space to 2 (xy) dimensions, t-SNE (t-distributed stochastic neighbor embedding) was used due to its ability to capture non-linear similarities in the high-dimensional space and reflect them in the low-dimensional space.^36^ The resulting plot is shown in Fig. 6.

Fig. 6 illustrates the t-SNE plot using all the measured values of the 49 experiments from the three independent campaigns. The markers are shaped according to the alkyne group of the experiment, and the annotations refer to the solvent, iodine source and temperature of the experiment. Green markers correspond to converged experiments surpassing a conversion of 80%.

The annotations of Fig. 6 help visualize the way that t-SNE reduced the parameter space into 2 dimensions. We see that the experiments are mainly clustered by their solvent and temperature. The vertical half is characterized by high temperatures and the lower half by low temperatures. The solvent clusters can be read from left to right as follows: DCM, DCE, DMSO, CH_3_CN/H_2_O, CH_3_CN and MeOH.

Overall, the best performing region of the contour locates at the right-hand side, and the majority of the converged points locate here. We can see more clearly here that none of the NIS experiments had high yields, and most of them concentrate on the left-hand side (poor performance) except the high-conversion point at higher temperature and CH_3_CN/H_2_O as shown in Fig. 5. Overall, the low-temperature chloramine route was preferred and shown to be more robust across reagents, solvents and temperatures.

The dimensionality reduction contour plot is also useful to visualize the optimization trajectory. Fig. 7 illustrates the sequence of experiments for the three optimization campaigns under the same dimensionality reduction as for Fig. 6. The color-code in Fig. 7 corresponds to the model predictions using only their individual campaign data as training, as opposed to Fig. 6 which shows all the experimental points color-coded by their true value. In Fig. 7, the evaluated experiments of each campaign are annotated with the iteration number at which they were visited, whereas the unlabeled open markers are only predicted and shown for comparison purposes with the ground-truth. Although the first iteration composed of 11 experiments is equivalent for all 3 algorithms, each of the optimization campaigns seems to exploit a particular region of the space and leverage this information for the remaining alkyne groups. For instance, the Falcon Light trajectory shows that the first iterations concentrate on the top left region for all three campaigns, but after discovering the MeOH solvent at iteration 3 and the TBAI reagent at iteration 4, the algorithm exploits this information to optimize the remaining alkyne groups (1-chloro-2-ethynylbenzene and 2-ethynyltoluene). Similar analysis can be done on the other two algorithms: Falcon DNGO concentrates on the bottom left and center regions (CH_3_CN/H_2_O|NaI/KI|25 °C), and Falcon GPBO concentrates on the top central region (MeOH solvent at higher temperature).

The t-SNE plots also permit comparing the predictive behavior of the different Falcon algorithms. When placing side-by-side the merit contour map of Fig. 6 – with the ground-truth merit values – with each of the contour maps of Fig. 7 – using only the campaign points as training data – it is possible to qualitatively compare the predictions of each of the algorithms with the real values by comparing the color schemes. This comparison reveals the deviated predictions in the unexplored regions for each of the algorithms. For example, while Falcon DNGO captures part of the local optimum around the top right of the contour plot, it mistakenly predicts a poor performance around the MeOH solvent and high temperature region for 2-ethynyltoluene, whereas this is an optimal region found by Falcon GPBO. For Falcon GPBO, the predicted merit follows a similar trend to that of Fig. 6, yet it mistakenly predicts a local optimum region at the bottom left (DCM solvent and NaI reagent for 2-ethynyltoluene). Falcon Light also identifies the optimality region at the top right close to the observed points yet fails to identify the optimal region at the bottom center. Qualitatively speaking, the surrogate of Falcon GPBO seems to capture the overall trend more accurately than the other algorithms given the explored points of each campaign. Gaussian processes are known to provide a good predictive power in the low-data regime, and visually the explored points of Falcon GPBO are more spread out in the space than for the other algorithms. Overall, the t-SNE plot can be used to observe mean-value predictions, uncertainty or acquisition function values of Bayesian optimizers to better explain their “reasoning” for experiment planning.

All the algorithms reached the goal of surpassing a conversion of 80% with Falcon GPBO and Falcon Light at 23 experiments (6 iterations) and Falcon DNGO at 25 experiments (7 iterations), which are a fraction of the total number of 12 036 combinations. Considering the 8-dimensional parameter space, converging in 23–25 experiments (0.2% of the search space) and 6–7 BO loops is consistent with other referenced BO applications,^9,33,34^ showcasing the robust applicability of these methods to different chemistry challenges.

These algorithms leverage the observed data to carefully select the next points to evaluate, thereby augmenting the sample efficiency in large and constrained search spaces. While two reaction paths were available, all the algorithms selected the chloramine path as it proved to be more robust to the tested reagents, solvents and temperatures.

## 4 Conclusions

We have successfully showcased the integration of robotic automation with Bayesian optimization techniques to explore the optimal iodination of terminal alkynes, achieved through the seamless integration of two cloud-based solutions: Atinary Technologies' ML engine, SDLabs, and IBM RoboRXN's robotic platform. We established a smooth workflow by creating a backend application that enabled communication between the two cloud-hosted systems using a JSON-based protocol to transfer reaction parameters and results. This setup allows for the optimization of reaction parameters characterizing synthesis procedures. Our solution demonstrates the potential for optimizing chemical synthesis in research and development against different routes and control parameters.

Our study focused on the iodination of terminal alkynes, which holds significant importance in synthesizing complex organic molecules while preserving the alkyl group and introducing an iodide group. By exploring two different routes, one involving chloramine salts and the other utilizing *N*-iodosuccinimide (NIS) as the iodinating agent, we sought to determine the most effective reaction conditions from a total of 12 036 possible combinations. Through the use of three distinct BO optimizers, we achieved impressive results, with all optimizers reaching over 80% conversion for all four reactants within a minimal number of just 25 distinct reactions, covering only *ca.* 0.2% of the total number of possible combinations. These findings showcase promising opportunities for efficient and highly productive iodination of terminal alkynes.

Finally, we took a first step towards tackling the interpretability issues of BO models through the implementation of diverse visualization techniques. Specifically, the incorporation of a customized contour plot based on t-SNE dimensionality reduction proved to be instrumental in improving the comprehension of BO methods. This novel approach effectively illuminates the otherwise opaque nature of BO algorithms, rendering them more transparent and insightful as an integral part of the self-driving labs platform. By addressing the challenge of interpretability, our research makes a valuable contribution to the wider acceptance and utilization of BO methods in chemical synthesis and other research and development endeavors.

In conclusion, we believe that the integration of ML-driven experiment planning, automated equipment, and interpretability tools has the potential to change the way we identify optimal synthesis routes and conditions, allowing for a cleaner and more sustainable industry.

## Data availability

Data for this paper, including the parameter grid and the measured yields, are available at https://10.5281/zenodo.10022311.

## Author contributions

Oliver Schilter: methodology, investigation, software, resources, conceptualization, visualization, data curation, writing – original draft, writing – review & editing. Daniel Pacheco Gutierrez: data curation, formal analysis, visualization, writing – original draft, software, writing – review & editing. Linnea M. Folkmann: software, data curation. Alessandro Castrogiovanni: methodology, resources, conceptualization. Alberto Garcia-Duran: conceptualization, software, visualization. Federico Zipoli: methodology, conceptualization, validation, visualization, data curation, writing – original draft, writing – review & editing. Loïc M. Roch: methodology, conceptualization, writing – original draft, writing – review & editing, resources, validation, project administration, supervision. Teodoro Laino: methodology, writing – original draft, writing – review & editing, project administration, supervision.

## Conflicts of interest

There are no conflicts to declare.

## Supplementary Material

SC-015-D3SC05607D-s001

SC-015-D3SC05607D-s002

SC-015-D3SC05607D-s003

SC-015-D3SC05607D-s004

SC-015-D3SC05607D-s005

SC-015-D3SC05607D-s006

SC-015-D3SC05607D-s007

SC-015-D3SC05607D-s008

SC-015-D3SC05607D-s009

SC-015-D3SC05607D-s010

SC-015-D3SC05607D-s011

SC-015-D3SC05607D-s012

SC-015-D3SC05607D-s013

SC-015-D3SC05607D-s014

SC-015-D3SC05607D-s015

SC-015-D3SC05607D-s016

SC-015-D3SC05607D-s017

SC-015-D3SC05607D-s018

SC-015-D3SC05607D-s019

SC-015-D3SC05607D-s020

SC-015-D3SC05607D-s021

SC-015-D3SC05607D-s022

SC-015-D3SC05607D-s023

SC-015-D3SC05607D-s024

SC-015-D3SC05607D-s025

SC-015-D3SC05607D-s026

## References

[cit1] Tackling Global Challenges, https://www.rsc.org/policy-evidence-campaigns/environmental-sustainability/global-challenges/, accessed: 2023-07-28

[cit2] Global Challenges, Chemistry Solutions, https://www.rsc.org/news-events/features/2015/jan/global-challenges-chemistry-solutions/, accessed: 2023-07-28

[cit3] Tabor D. P., Roch L. M., Saikin S. K., Kreisbeck C., Sheberla D., Montoya J. H., Dwaraknath S., Aykol M., Ortiz C., Tribukait H. (2018). et al.. Nat. Rev. Mater..

[cit4] Butler K. T., Davies D. W., Cartwright H., Isayev O., Walsh A. (2018). Nature.

[cit5] Gomes C. P., Selman B., Gregoire J. M. (2019). MRS Bull..

[cit6] Sha W., Guo Y., Yuan Q., Tang S., Zhang X., Lu S., Guo X., Cao Y.-C., Cheng S. (2020). Adv. Intell. Syst..

[cit7] Aspuru-Guzik A. (2020). Artif. Intell. Drug Discovery.

[cit8] Suh C., Fare C., Warren J. A., Pyzer-Knapp E. O. (2020). Annu. Rev. Mater. Res..

[cit9] Shields B. J., Stevens J., Li J., Parasram M., Damani F., Alvarado J. I. M., Janey J. M., Adams R. P., Doyle A. G. (2021). Nature.

[cit10] Guo J., Ranković B., Schwaller P. (2023). Chimia.

[cit11] Christensen M., Yunker L. P., Adedeji F., Häse F., Roch L. M., Gensch T., dos Passos Gomes G., Zepel T., Sigman M. S., Aspuru-Guzik A. (2021). et al.. Commun. Chem..

[cit12] Braconi E., Godineau E. (2023). ACS Sustain. Chem. Eng..

[cit13] Taylor C. J., Pomberger A., Felton K. C., Grainger R., Barecka M., Chamberlain T. W., Bourne R. A., Johnson C. N., Lapkin A. A. (2023). Chem. Rev..

[cit14] Toniato A., Schilter O., Laino T. (2023). Chimia.

[cit15] Pyzer-Knapp E. O., Pitera J. W., Staar P. W., Takeda S., Laino T., Sanders D. P., Sexton J., Smith J. R., Curioni A. (2022). npj Comput. Mater..

[cit16] Friedman M. and Savage L. J., Techniques of Statistical Analysis, 1947, pp. 365–372

[cit17] Daniel C. (1973). J. Am. Stat. Assoc..

[cit18] Box J. F. (1980). Am. Stat..

[cit19] Gutierrez D. P., Folkmann L. M., Tribukait H., Roch L. M. (2023). Chimia.

[cit20] Johnson P. C., Laurell C., Ots M., Sandström C. (2022). Technol. Forecast. Soc. Change.

[cit21] Krenn M., Pollice R., Guo S. Y., Aldeghi M., Cervera-Lierta A., Friederich P., dos Passos Gomes G., Häse F., Jinich A., Nigam A. (2022). et al.. Nat. Rev. Phys..

[cit22] Pollice R., dos Passos Gomes G., Aldeghi M., Hickman R. J., Krenn M., Lavigne C., Lindner-D'Addario M., Nigam A., Ser C. T., Yao Z. (2021). et al.. Acc. Chem. Res..

[cit23] França C. , arXiv, 2023, preprint, arXiv:2307.10265, 10.48550/arXiv.2307.10265

[cit24] Mockus J. B. , The Bayesian Approach to Global Optimization, Freie Univ., Fachbereich Mathematik, 1984

[cit25] Nikolaev P., Hooper D., Webber F., Rao R., Decker K., Krein M., Poleski J., Barto R., Maruyama B. (2016). npj Comput. Mater..

[cit26] Dragone V., Sans V., Henson A. B., Granda J. M., Cronin L. (2017). Nat. Commun..

[cit27] Kitson P. J., Marie G., Francoia J.-P., Zalesskiy S. S., Sigerson R. C., Mathieson J. S., Cronin L. (2018). Science.

[cit28] Zhou Z., Li X., Zare R. N. (2017). ACS Cent. Sci..

[cit29] Xue D., Balachandran P. V., Hogden J., Theiler J., Xue D., Lookman T. (2016). Nat. Commun..

[cit30] Duros V., Grizou J., Xuan W., Hosni Z., Long D.-L., Miras H. N., Cronin L. (2017). Angew. Chem..

[cit31] MacLeod B. P., Parlane F. G., Morrissey T. D., Häse F., Roch L. M., Dettelbach K. E., Moreira R., Yunker L. P., Rooney M. B., Deeth J. R. (2020). et al.. Sci. Adv..

[cit32] Langner S., Häse F., Perea J. D., Stubhan T., Hauch J., Roch L. M., Heumueller T., Aspuru-Guzik A., Brabec C. J. (2020). Adv. Mater..

[cit33] Taylor C. J., Felton K. C., Wigh D., Jeraal M. I., Grainger R., Chessari G., Johnson C. N., Lapkin A. A. (2023). ACS Cent. Sci..

[cit34] Clayton A. D., Pyzer-Knapp E. O., Purdie M., Jones M. F., Barthelme A., Pavey J., Kapur N., Chamberlain T. W., Blacker A. J., Bourne R. A. (2023). Angew. Chem., Int. Ed..

[cit35] Moosbauer J. , Casalicchio G., Lindauer M. and Bischl B., arXiv, 2022, preprint, arXiv:2206.05447, 10.48550/arXiv.2206.05447

[cit36] Van der Maaten L., Hinton G. (2008). J. Mach. Learn. Res..

[cit37] McInnes L. , Healy J. and Melville J., arXiv, 2018, preprint, arXiv:1802.03426, 10.48550/arXiv.1802.03426

[cit38] Joswiak M., Peng Y., Castillo I., Chiang L. H. (2019). Control Eng. Pract..

[cit39] Trozzi F., Wang X., Tao P. (2021). J. Phys. Chem. B.

[cit40] Häse F., Roch L. M., Aspuru-Guzik A. (2019). Trends Chem..

[cit41] Roch L. M., Häse F., Kreisbeck C., Tamayo-Mendoza T., Yunker L. P., Hein J. E., Aspuru-Guzik A. (2018). Sci. Robot..

[cit42] Laveille P., Miéville P., Chatterjee S., Clerc E., Cousty J.-C., de Nanteuil F., Lam E., Mariano E., Ramirez A., Randrianarisoa U. (2023). et al.. Chimia.

[cit43] CAPeX: Pioneer Center for Accelerating P2X Materials Discovery, https://capex.dtu.dk/, accessed: 2024-02-29

[cit44] Polybot: New Autonomous Discovery Platform Built in the Center for Nanoscale Materials., https://www.anl.gov/cnm/polybot, accessed: 2024-02-29

[cit45] High-Throughput Experimentation Laboratory, https://www.chem.uzh.ch/en/research/services/htel.html, accessed: 2024-02-29

[cit46] What are Self-Driving Labs and How are They Transforming the Chemical Industry?, https://www.weforum.org/agenda/2024/01/self-driving-labs-transforming-chemical-industry/, accessed: 2024-02-29

[cit47] Ramirez A., Lam E., Gutierrez D. P., Hou Y., Tribukait H., Roch L. M., Copéret C., Laveille P. (2024). Chem Catal..

[cit48] Burger B., Maffettone P. M., Gusev V. V., Aitchison C. M., Bai Y., Wang X., Li X., Alston B. M., Li B., Clowes R. (2020). et al.. Nature.

[cit49] Zeng W., Wu W., Jiang H., Sun Y., Chen Z. (2013). Tetrahedron Lett..

[cit50] Chen Z., Jiang H., Wang A., Yang S. (2010). J. Org. Chem..

[cit51] Villeneuve K., Riddell N., Jordan R. W., Tsui G. C., Tam W. (2004). Org. Lett..

[cit52] Shi W., Lei A. (2014). Tetrahedron Lett..

[cit53] Chen Z., Zeng W., Jiang H., Liu L. (2012). Org. Lett..

[cit54] Yao M., Zhang J., Yang S., Liu E., Xiong H. (2020). Synlett.

[cit55] Liu X., Chen G., Li C., Liu P. (2018). Synlett.

[cit56] Liu H., Wei Y., Cai C. (2016). Synlett.

