# Supplementary material for: Combining Bayesian optimization and automation to simultaneously optimize reaction conditions and routes
Source: Chem Sci. 2024 Apr 29;15(20):7732–41. doi: 10.1039/d3sc05607d (PMC11110165; doi:10.1039/d3sc05607d)
Supplement: SC-015-D3SC05607D-s001 [file SC-015-D3SC05607D-s001.pdf]

Electronic Supplementary Material (ESI) for Chemical Science.
This journal is © The Royal Society of Chemistry 2024

Electronic Supporting Information: Combining Bayesian optimization and automation to simultaneously optimize reaction conditions and routes

Oliver Schilter*[a,b], Daniel Pacheco Gutierrez[c], Linnea M. Folkmann[c], Alessandro Castrogiovanni[a], Alberto García-Durán[c], Federico Zipoli[a,b], Loïc M. Roch[c] and Teodoro Laino[a,b]

A Supporting Material

A.1 T-SNE plot description

t-SNE, or t-Distributed Stochastic Neighbor Embedding is a dimensionality-reduction technique used to visualize high-dimensional data. It converts the high-dimensional Euclidean distances between data points into Gaussian conditional probabilities that represent similarities. Points that are close together in high-dimensional space have a higher probability of being picked than points that are far apart. The goal is to accomplish a similar probability distribution in the low-dimensional space. The probability distribution in the low-dimensional space is modeled as a Student T Distribution with 1 degree of freedom. The algorithm then minimizes the Kullback-Leibler divergence between these two distributions by fine-tuning the locations of the points in the low-dimensional space. It must be noted that as this algorithm tries to match conditional probabilities of neighbors, the distance between clusters does not necessarily relate to distances in the high-dimensional spaces. Also, clusters in low-dimensional spaces don't imply clusters in high dimensional spaces. This method is a visualization technique that guarantees that neighboring points are similar. An important parameter for this technique is the perplexity, which relates to the number of neighbors associated to a given point, and typical values range between 5 and 50 and should be fine-tuned to generate a sensible representation.

[a] *IBM Research Europe, Säumerstrasse 4, 8803 Rüschlikon, Switzerland. E-mail: oli@zurich.ibm.com*
[b] *National Center for Competence in Research-Catalysis (NCCR-Catalysis), Switzerland*
[c] *Atinary Technologies, Route de la Corniche 4, 1066 Epalinges, Switzerland*

A.2 Scatter plots: All algorithms

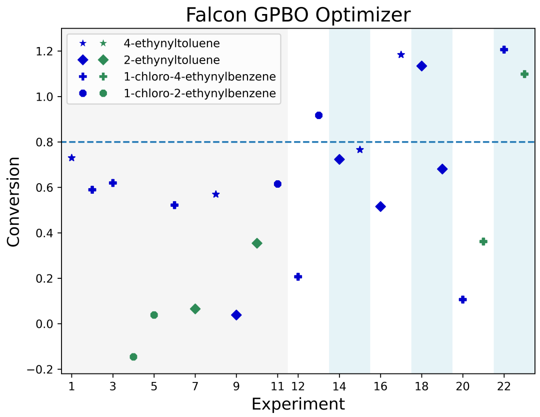

Fig. A.1 Conversion Progress chart for the Falcon GPBO optimizer. The gray area corresponds to the initial 11 reactions. Each pair of parameters is marked with a change in background color, the shape of the dots indicates the alkyne used, while the color indicates if the NIS (green) or cloramine (blue) route was used. If an alkyne is converted over 80% (dotted line) the alkyne is no longer further optimized. The optimization campaign is ended when all four substrates reach 80% conversion

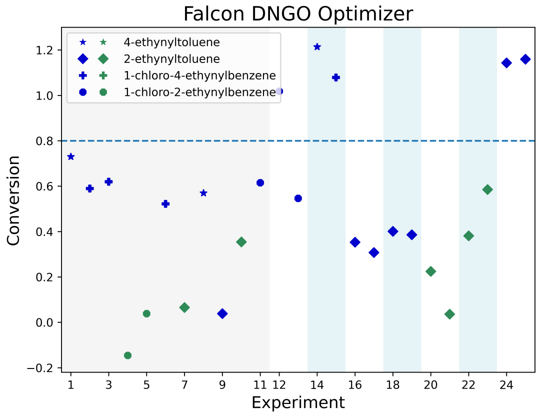

Fig. A.2 Conversion progress chart for the Falcon DNGO optimizer. The gray area corresponds to the initial 11 reactions. Each pair of parameters is marked with a change in background color, the shape of the dots indicates the alkyne used, while the color indicates if the NIS (green) or cloramine (blue) route was used. If an alkyne is converted over 80% (dotted line) the alkyne is no longer further optimized. The optimization campaign is ended when all four substrates reach 80% conversion

A.3 T-SNE plots

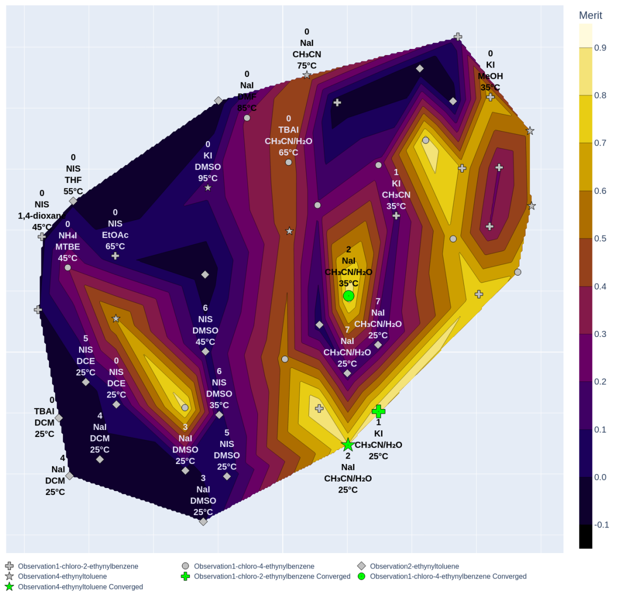

Fig. A.3 t-SNE plot for Falcon DNGO. The marker annotations correspond to the iteration number of the specific campaign. The color-code corresponds to the predicted surrogate model using only the labelled markers as training data.

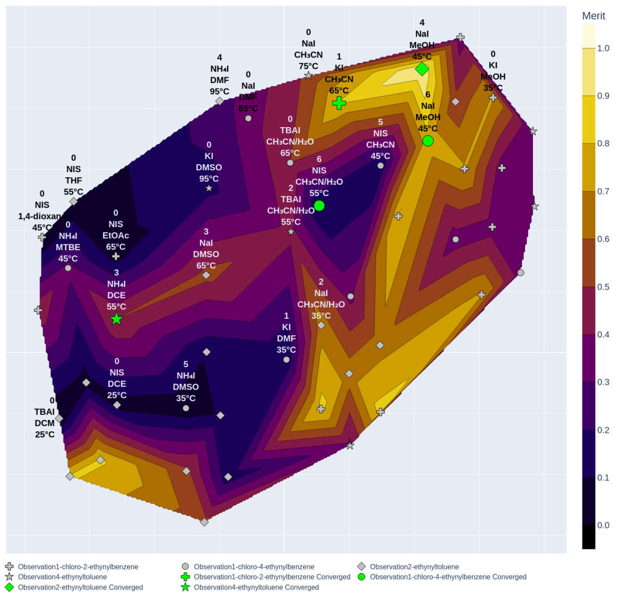

Fig. A.4 t-SNE plot for Falcon GPBO. The marker annotations correspond to the iteration number of the specific campaign. The color-code corresponds to the predicted surrogate model using only the labelled markers as training data.

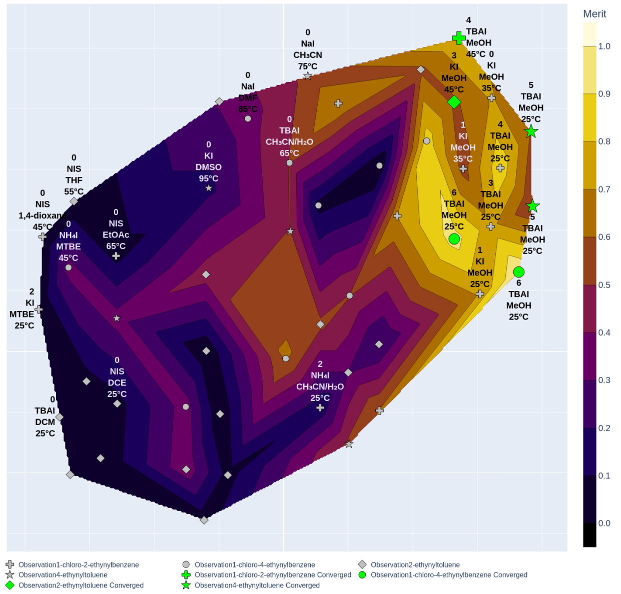

Fig. A.5 t-SNE plot for Falcon LIGHT. The marker annotations correspond to the iteration number of the specific campaign. The color-code corresponds to the predicted surrogate model using only the labelled markers as training data.

A.4 Example synthesis procedure executed by the robot

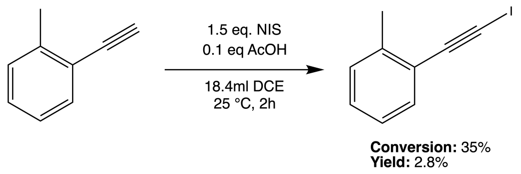

Fig. A.6 Iodination of 2-ethynyltoluene

To a 100ml stainless steel reactor, N-Iodosuccinimide (1.5 mmol, 337.4mg, 1.5eq) was added. The reactor was sealed and placed into the robotic platform and vacuum nitrogen was flushed three times. The robotic arm transferred 18.42ml of 1,2-Dichlorethan from a glass vial to the reactor using a syringe pump. The syringe and needle were automatically rinsed three times using isopropanol before the robotic arm added 1ml of 1M 2-ethynyltoluene solution in acetonitrile (1ml, 1M, 1eq, 1mmol). This solution was prepared by mixing 2-ethynyltoluene (1.16g, 0.01mol) in 10ml acetonitrile. After rinsing the syringe and needle the robotic platform added Acetic acid (0.57ml, 0.1mmol, 0.1eq.) to reach an overall reaction volume of 20ml. The mixture then was stirred for 2h at 25C°. The reaction mixture was quenched by adding saturated sodium thiosulfate (1ml) and cooling/keeping the reaction mixture a room temperature (25°C). As an internal standard 1ml of 1M acetophenon in acetonitrile solution (1mmol, 1eq) was added to the reactor by the robotic arm. A 0.3ml sample of the reaction mixture was diluted by the robotic arm 50 times with acetonitrile before filtering it. A 0.3μl sample was injected into a loop connecting the robotic platform to an HPLC/MS setup. After the mixture was separated through the reverse phase column (Agilent ZORBAX Eclipse Plus C18) each

area of the peak was integrated and analyzed. The area of the peak of the internal standard was utilized to validate that the injection was performed correctly and to normalize the areas of the product or starting material. Using an external calibration a conversion of 35% and a yield of 2.8% was determined.

A.5 Reference synthesis of iodoalkynes for characterization

A.5.1 1-(iodoethynyl)-2-methylbenzene

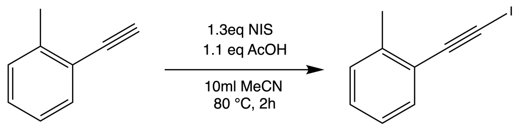

Fig. A.7 Iodination of 2-ethynyltoluene

To a 25ml glassflask 1.26ml 2-ethynyltoluene (1.16g, 10mmol, 1eq) was added and diluted in 10ml acetonitrile. To this solution N-Iodosuccinimide (11mmol, 2.47g, 1.1eq) and acetic acid (1.3ml, 13mmol, 1.3eq) were added. The mixture was stirred at 80°C for 2h before letting it cool to room temperature and extracted with 10ml saturated aqueous thiosulfate solution and 10ml ethyl acetate three times. The organic phase was collected and concentrated under vacuum, and the resulting crude oil was purified using reverse phase chromatography using a C-18 column with a gradient of mixture of water:acetonitrile of 50%:50% to 30%:70%. The product was analyzed via NMR spectroscopy: ^{1}H-NMR ($CDCl_3$, 80 MHz): δ (ppm) 7.35 (m, 4H), 2.12 (s, 3H);

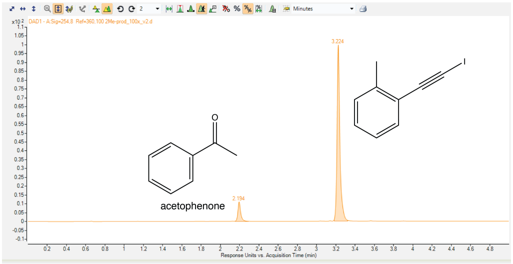

Fig. A.8 HPLC-DAD spectrum of the resulting product and the internal standard (acetophenone)

A.5.2 1-(iodoethynyl)-4-methylbenzene

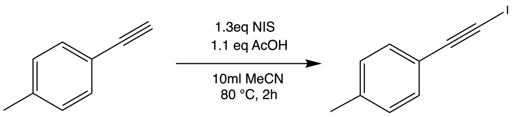

Fig. A.9 Iodination of 4-ethynyltoluene

To a 25ml glassflask 1.26ml 4-ethynyltoluene (1.16g, 10mmol, 1eq) was added and diluted in 10ml acetonitrile. To this solution N-Iodosuccinimide (11mmol, 2.47g, 1.1eq) and acetic acid (1.3ml, 13mmol, 1.3eq) were added. The mixture was stirred at 80°C for 2h before letting it cool to room temperature and extracted with 10ml saturated aqueous thiosulfate solution and 10ml ethyl acetate three times. The organic phase was collected and concentrated under vacuum, and the resulting crude oil was purified using silica gel chromatography with n-hexane as solvent. The resulting pale yellow oil was analyzed via NMR spectroscopy: ^{1}H-NMR ($CDCl_3$, 80 MHz): δ (ppm) 7.43 (m, 4H), 2.36 (s, 3H); ^{13}C-NMR($CDCl_3$, 80 MHz): δ (ppm) 138.99, 132.19, 128.97, 120.46, 94.24,80.21, 21.53, 5.01; the spectroscopic data are in agreement with assigned structures and consistent with literature[1].

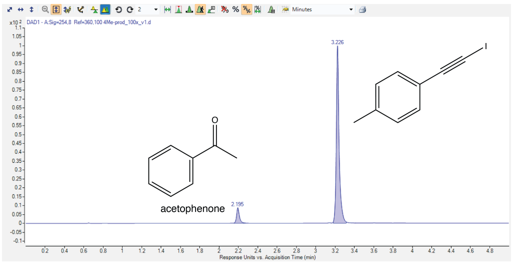

Fig. A.10 HPLC-DAD spectrum of the resulting product and the internal standard (acetophenone)

A.5.3 1-chloro-2-(iodoethynyl)benzene

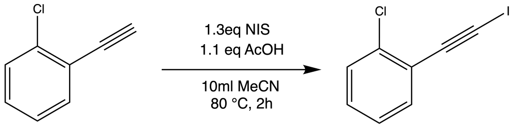

Fig. A.11 Iodination of 1-chloro-2-ethynylbenzene

To a 25ml glassflask 1.21ml 1-chloro-2-ethynylbenzene (1.36g, 10mmol, 1eq) was added and diluted in 10ml acetonitrile. To this solution N-Iodosuccinimide (11mmol, 2.47g, 1.1eq) and acetic acid (1.3ml, 13mmol, 1.3eq) were added. The mixture was stirred at 80°C for 2h before letting it cool to room temperature and extracted with 10ml saturated aqueous thiosulfate solution and 10ml ethyl acetate three times. The organic phase was collected and concentrated under vacuum, and the resulting crude oil was purified using reverse phase chromatography using a C-18 column with a gradient of mixture of water:acetonitrile of 50%:50% to 30%:70%. The resulting clear oil was analyzed via NMR spectroscopy: ^{1}H-NMR ($CDCl_3$, 80 MHz): δ (ppm) 7.43 (m, 4H); ^{13}C-NMR($CDCl_3$, 80 MHz): δ (ppm) 134.10, 130.10, 129.68, 129.43, 128.15, 127.15, 126.28, 84.70; the spectroscopic data are in agreement with assigned structures and consistent with literature[1].

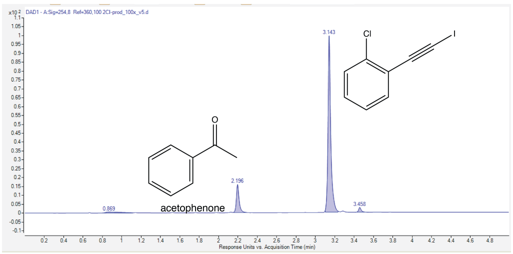

Fig. A.12 HPLC-DAD spectrum of the resulting product and the internal standard (acetophenone)

A.5.4 1-chloro-4-(iodoethynyl)benzene

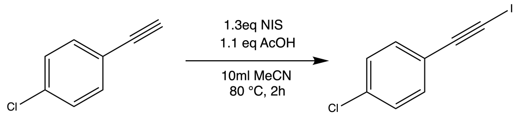

Fig. A.13 Iodination of 1-chloro-4-ethynylbenzene

To a 25ml glassflask 1.36g 1-chloro-4-ethynylbenzene (1.36g, 10mmol, 1eq) was added and diluted in 10ml acetonitrile. To this solution N-Iodosuccinimide (11mmol, 2.47g, 1.1eq) and acetic acide (1.3ml, 13mmol, 1.3eq) were added. The mixture was stirred at 80°C for 2h before letting it cool to room temperature and extracted with 10ml saturated aqueous thiosulfate solution and 10ml ethyl acetate three times. The organic phase was collected and concentrated under vacuum, and the resulting crude oil was purified using reverse phase chromatography using a C-18 column with a gradient of mixture of water:acetonitrile of 50%:50% to 30%:70%. The resulting white powder was filtered and was analyzed via NMR spectroscopy: ^{1}H-NMR (CDCl$_3$, 80 MHz): δ (ppm) 7.31 (m, 4H), 1.62 (H_2O); the spectroscopic data are in agreement with assigned structures and consistent with literature[2].

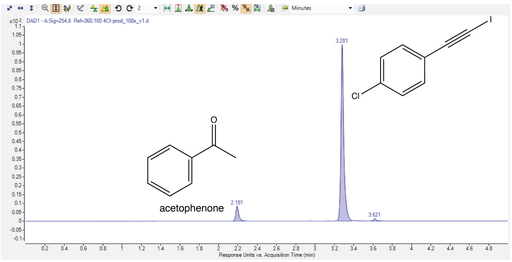

Fig. A.14 HPLC-DAD spectrum of the resulting product and the internal standard (acetophenone)

A.6 Calibration curves

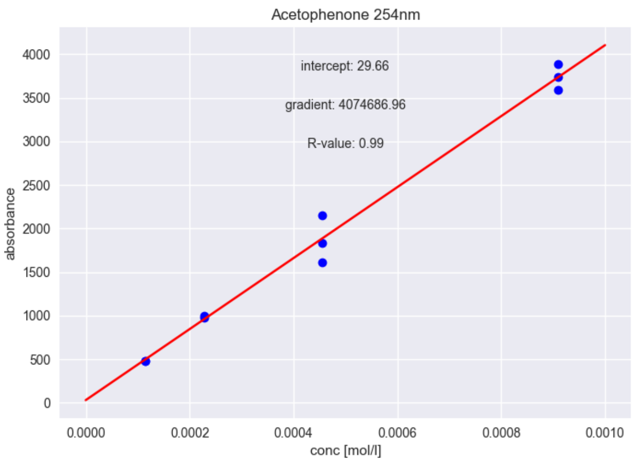

Fig. A.15 Calibration curve, where the peak area is plotted against the known concentration at 254nm.

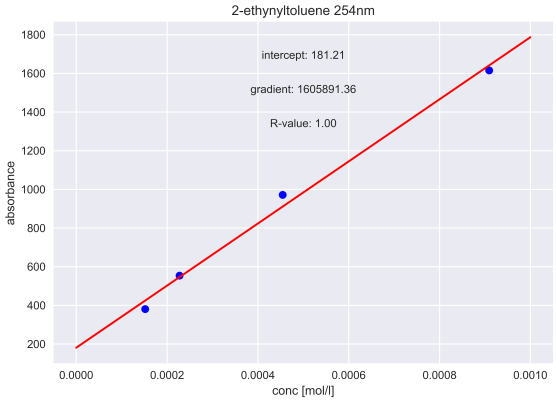

Fig. A.16 Calibration curve, where the peak area is plotted against the known concentration at 254nm.

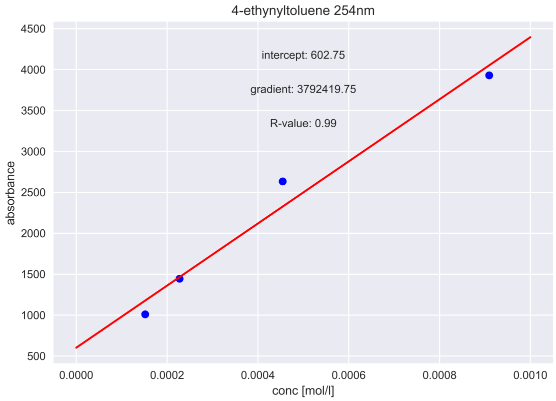

Fig. A.17 Calibration curve, where the peak area is plotted against the known concentration at 254nm.

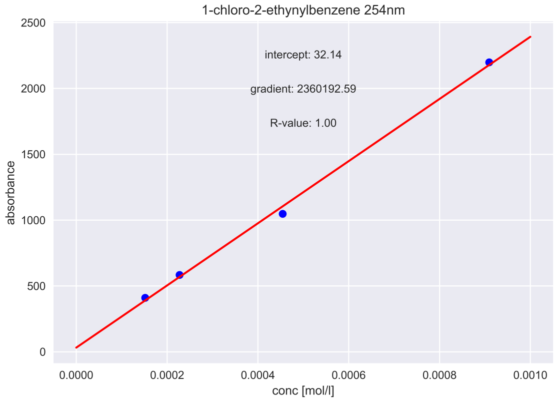

Fig. A.18 Calibration curve, where the peak area is plotted against the known concentration at 254nm.

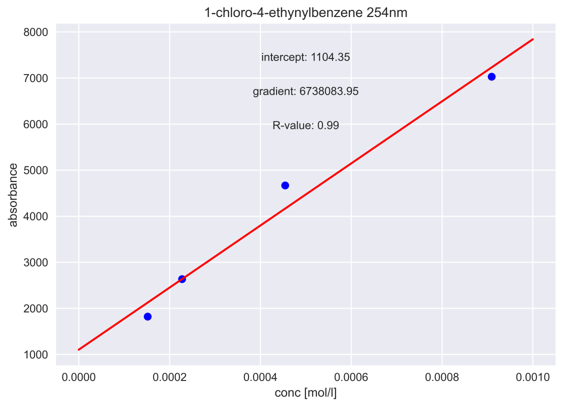

Fig. A.19 Calibration curve, where the peak area is plotted against the known concentration at 254nm.

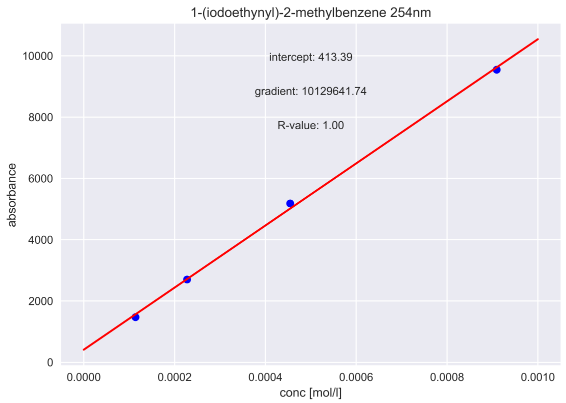

Fig. A.20 Calibration curve, where the peak area is plotted against the known concentration at 254nm.

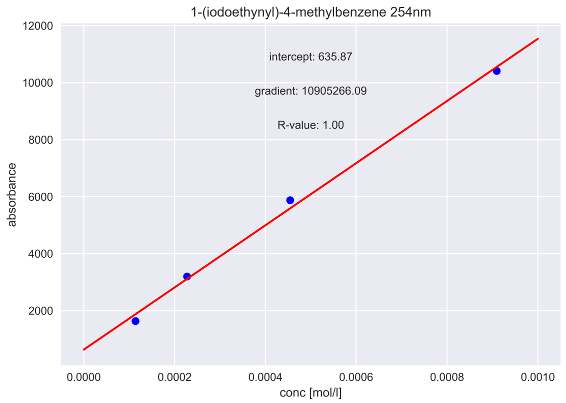

Fig. A.21 Calibration curve, where the peak area is plotted against the known concentration at 254nm.

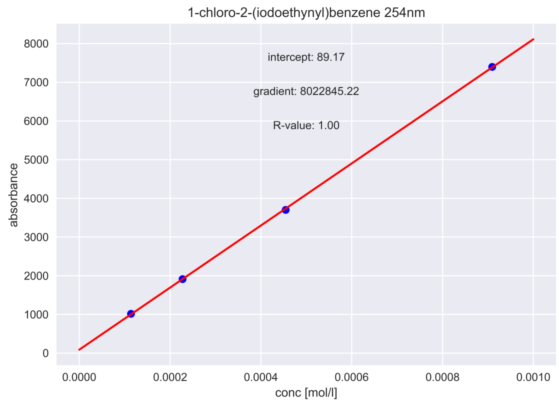

Fig. A.22 Calibration curve, where the peak area is plotted against the known concentration at 254nm.

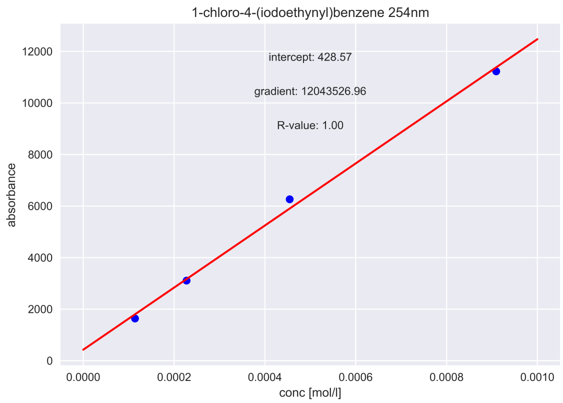

Fig. A.23 Calibration curve, where the peak area is plotted against the known concentration at 254nm.

Notes and references

1 X. Liu, G. Chen, C. Li and P. Liu, *Synlett*, 2018, **29**, 2051–2055.
2 M. Yao, J. Zhang, S. Yang, E. Liu and H. Xiong, *Synlett*, 2020, **31**, 1102–1106.

A.7 Parallel Coordinates

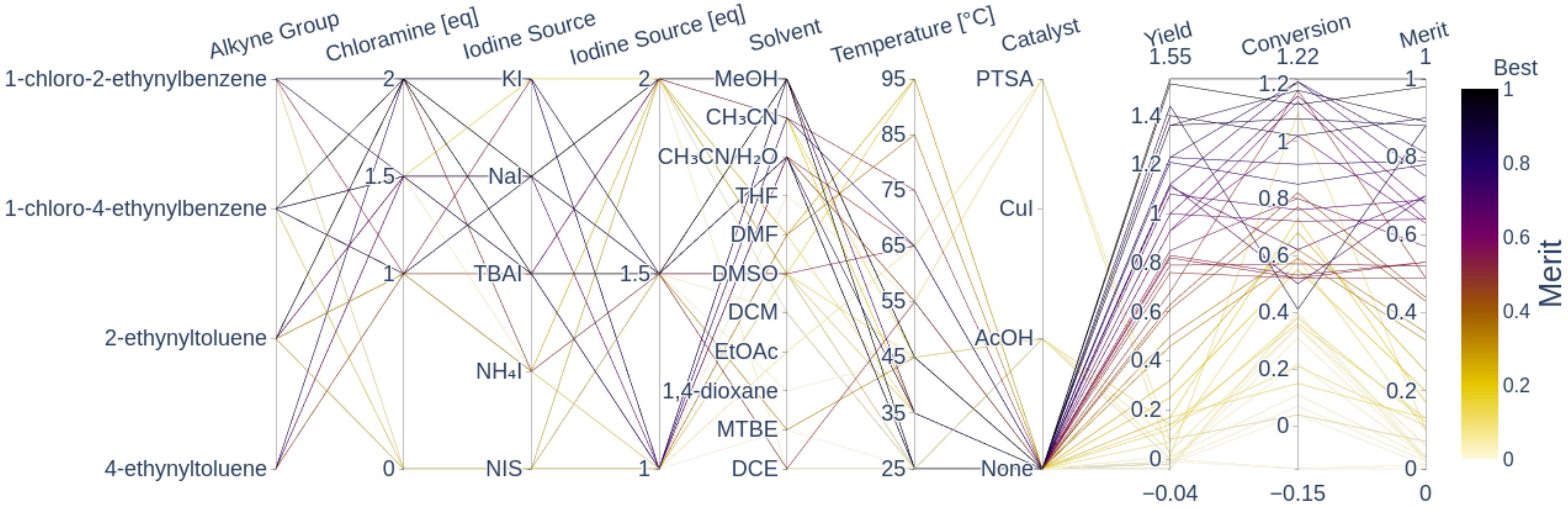

Fig. A.24 Parallel Coordinates of the 49 experiments from the three optimization campaigns. Each line corresponds to an experiment and the columns correspond to the different variables (parameters, objectives and weighted-sum merit). The highlighted traces correspond to the experiments with a conversion surpassing 80%. The merit corresponds to a normalized weighted sum; the higher the better the performance. The traces are color-coded by the merit (i.e. darker traces have better performance than lighter ones).

A.8 Reaction parameters

Table 1 Reaction conditions initial 11 reactions

Campaign	exp	Alkyne	Chloramine [eq]	Iodine src		Solvent	Temp [°C]	Catalyst	Yield	Conv	Merit
Initial	1	4-ethynyltoluene	1.5	KI	2.0	DMSO	95		0.17	0.73	0.83
Initial	2	1-chloro-4-ethynylbenzene	1.0	NH_4I	1.5	MTBE	45		0.46	0.59	0.67
Initial	3	1-chloro-4-ethynylbenzene	2.0	NaI	1.0	DMF	85		0.50	0.62	0.65
Initial	4	1-chloro-2-ethynylbenzene		NIS	1.5	1,4-dioxane	45	AcOH	0.00	-0.15	0.99
Initial	5	1-chloro-2-ethynylbenzene		NIS	1.0	EtOAc	65	PTSA	0.08	0.04	0.93
Initial	6	1-chloro-4-ethynylbenzene	1.5	TBAI	1.0	CH_3CN/H_2O	65		0.76	0.52	0.51
Initial	7	2-ethynyltoluene		NIS	1.0	THF	55		-0.01	0.07	0.98
Initial	8	4-ethynyltoluene	1.5	NaI	2.0	CH_3CN	75		0.79	0.57	0.48
Initial	9	2-ethynyltoluene	1.5	TBAI	2.0	DCM	25		-0.02	0.04	0.99
Initial	10	2-ethynyltoluene		NIS	1.5	DCE	25	AcOH	0.03	0.35	0.94
Initial	11	1-chloro-2-ethynylbenzene	2.0	KI	1.0	MeOH	35		1.11	0.62	0.30

Table 2 Reaction conditions of the Falcon DNGO algorithm

Campaign	exp	itt	Alkyne	Chloramine [eq]	Iodine src		Solvent	Temp [°C]	Catalyst	Yield	Conv	Merit
DNGO	12	1	1-chloro-2-ethynylbenzene	2.0	KI	1.5	CH_3CN/H_2O	25		1.40	1.02	0.10
DNGO	13	1	1-chloro-2-ethynylbenzene	2.0	KI	1.0	CH_3CN	35		0.25	0.55	0.80
DNGO	14	2	4-ethynyltoluene	2.0	NaI	1.5	CH_3CN/H_2O	25		1.23	1.21	0.19
DNGO	15	2	1-chloro-4-ethynylbenzene	1.0	NaI	1.5	CH_3CN/H_2O	35		1.36	1.08	0.12
DNGO	16	3	2-ethynyltoluene	1.0	NaI	2.0	DMSO	25		-0.02	0.35	0.97
DNGO	17	3	2-ethynyltoluene	2.0	NaI	1.5	DMSO	25		-0.02	0.31	0.97
DNGO	18	4	2-ethynyltoluene	1.0	NaI	1.5	DCM	25		-0.04	0.40	0.97
DNGO	19	4	2-ethynyltoluene	1.5	NaI	2.0	DCM	25		-0.04	0.39	0.98
DNGO	20	5	2-ethynyltoluene		NIS	2.0	DCE	25	AcOH	0.00	0.22	0.97
DNGO	21	5	2-ethynyltoluene		NIS	2.0	DMSO	25	AcOH	0.08	0.04	0.93
DNGO	22	6	2-ethynyltoluene		NIS	1.0	DMSO	45		0.12	0.38	0.88
DNGO	23	6	2-ethynyltoluene		NIS	1.5	DMSO	35		0.07	0.59	0.90
DNGO	24	7	2-ethynyltoluene	1.5	NaI	1.5	CH_3CN/H_2O	25		0.93	1.14	0.36
DNGO	25	7	2-ethynyltoluene	2.0	NaI	1.5	CH_3CN/H_2O	25		1.00	1.16	0.32

Table 3 Reaction conditions of the Falcon GPBO algorithm

Campaign	exp	itt	Alkyne	Chloramine [eq]	Iodine src		Solvent	Temp [°C]	Catalyst	Yield	Conv	Merit
GPBO	12	1	1-chloro-4-ethynylbenzene	1.0	KI	1.0	DMF	35		0.14	0.21	0.89
GPBO	13	1	1-chloro-2-ethynylbenzene	2.0	KI	1.0	CH_3CN	65		1.23	0.92	0.21
GPBO	14	2	2-ethynyltoluene	2.0	NaI	1.0	CH_3CN/H_2O	35		1.00	0.72	0.36
GPBO	15	2	4-ethynyltoluene	1.0	TBAI	1.0	CH_3CN/H_2O	55		0.61	0.77	0.57
GPBO	16	3	2-ethynyltoluene	2.0	NaI	1.5	DMSO	65		0.82	0.52	0.47
GPBO	17	3	4-ethynyltoluene	2.0	NH_4I	1.5	DCE	55		0.68	1.18	0.51
GPBO	18	4	2-ethynyltoluene	2.0	NaI	2.0	MeOH	45		1.53	1.13	0.02
GPBO	19	4	2-ethynyltoluene	1.0	NH_4I	2.0	DMF	95		0.32	0.68	0.75
GPBO	20	5	1-chloro-4-ethynylbenzene	1.5	NH_4I	1.0	DMSO	35		0.01	0.11	0.97
GPBO	21	5	1-chloro-4-ethynylbenzene		NIS	2.0	CH_3CN	45	AcOH	0.14	0.36	0.87
GPBO	22	6	1-chloro-4-ethynylbenzene	1.5	NaI	1.5	MeOH	45		1.12	1.21	0.25
GPBO	23	6	1-chloro-4-ethynylbenzene		NIS	1.5	CH_3CN/H_2O	55	PTSA	0.01	1.10	0.90

Table 4 Reaction conditions of the Falcon Light algorithm

Campaign	exp	itt	Alkyne	Chloramine [eq]	Iodine src		Solvent	Temp [°C]	Catalyst	Yield	Conv	Merit
LIGHT	12	1	1-chloro-2-ethynylbenzene	2.0	KI	1.0	MeOH	25		1.12	0.50	0.30
LIGHT	13	1	1-chloro-2-ethynylbenzene	1.0	KI	1.5	MeOH	35		0.83	0.53	0.47
LIGHT	14	2	1-chloro-2-ethynylbenzene	2.0	NH_4I	1.0	CH_3CN/H_2O	25		0.17	0.15	0.87
LIGHT	15	2	1-chloro-2-ethynylbenzene	2.0	KI	1.0	MTBE	25		-0.01	-0.15	1.00
LIGHT	16	3	1-chloro-2-ethynylbenzene	2.0	TBAI	1.0	MeOH	25		1.08	0.76	0.31
LIGHT	17	3	2-ethynyltoluene	2.0	KI	1.0	MeOH	45		0.85	0.80	0.43
LIGHT	18	4	1-chloro-2-ethynylbenzene	1.5	TBAI	1.5	MeOH	25		1.44	0.41	0.12
LIGHT	19	4	1-chloro-2-ethynylbenzene	2.0	TBAI	1.0	MeOH	45		1.21	0.85	0.22
LIGHT	20	5	4-ethynyltoluene	1.0	TBAI	2.0	MeOH	25		0.63	0.82	0.56
LIGHT	21	5	4-ethynyltoluene	1.5	TBAI	2.0	MeOH	25		0.93	1.02	0.37
LIGHT	22	6	1-chloro-4-ethynylbenzene	2.0	TBAI	1.5	MeOH	25		1.55	1.22	0.00
LIGHT	23	6	1-chloro-4-ethynylbenzene	1.5	TBAI	1.5	MeOH	25		1.36	1.18	0.11
