# Supplementary figures and images for: Combining Bayesian optimization and automation to simultaneously optimize reaction conditions and routes
Source: Chem Sci. 2024 Apr 29;15(20):7732–41. doi: 10.1039/d3sc05607d (PMC11110165; doi:10.1039/d3sc05607d)

1-chloro-2-ethynylbenzene 254nm

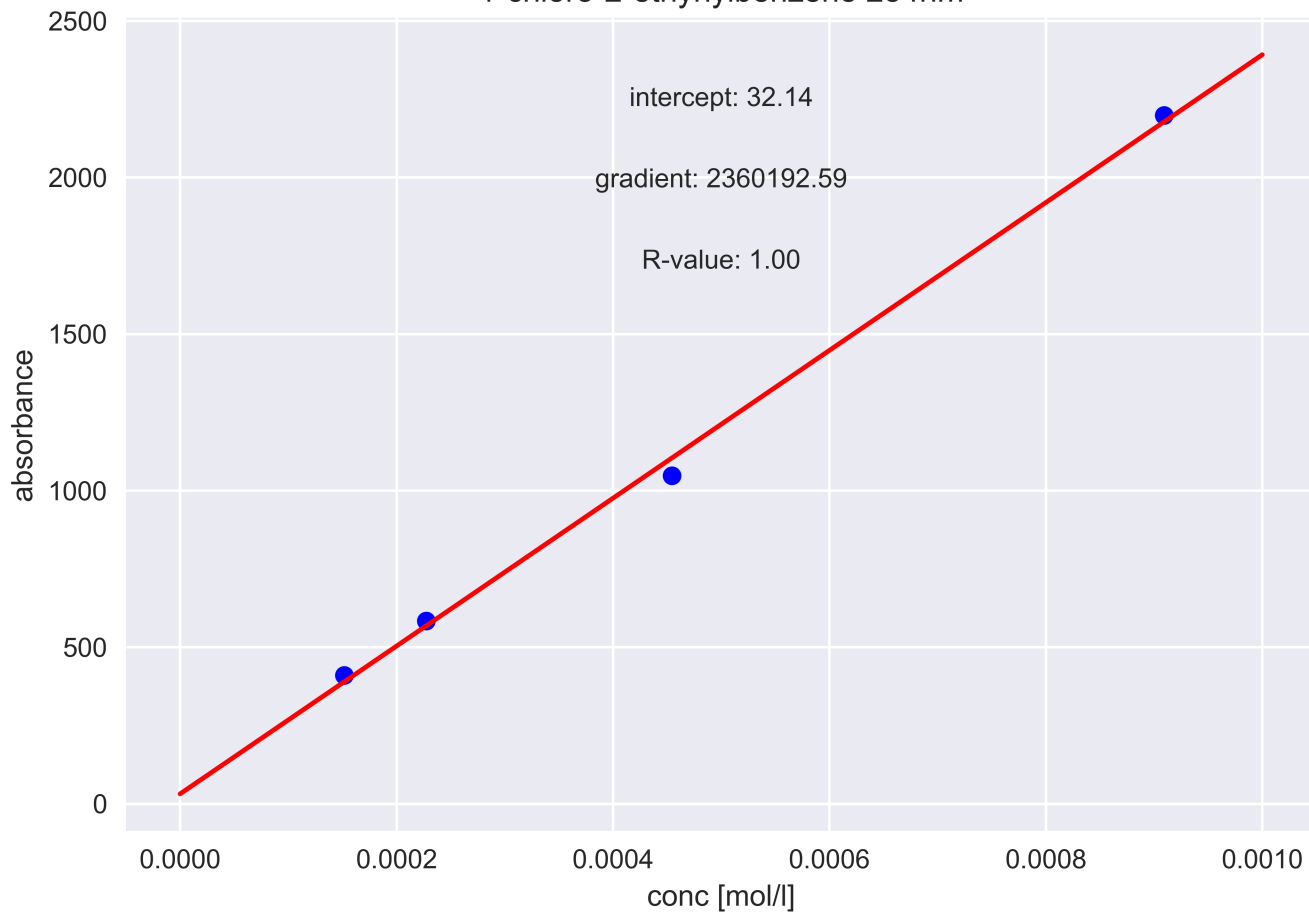

Supplement: SC-015-D3SC05607D-s002 [file SC-015-D3SC05607D-s002.pdf]

1-chloro-4-ethynylbenzene 254nm

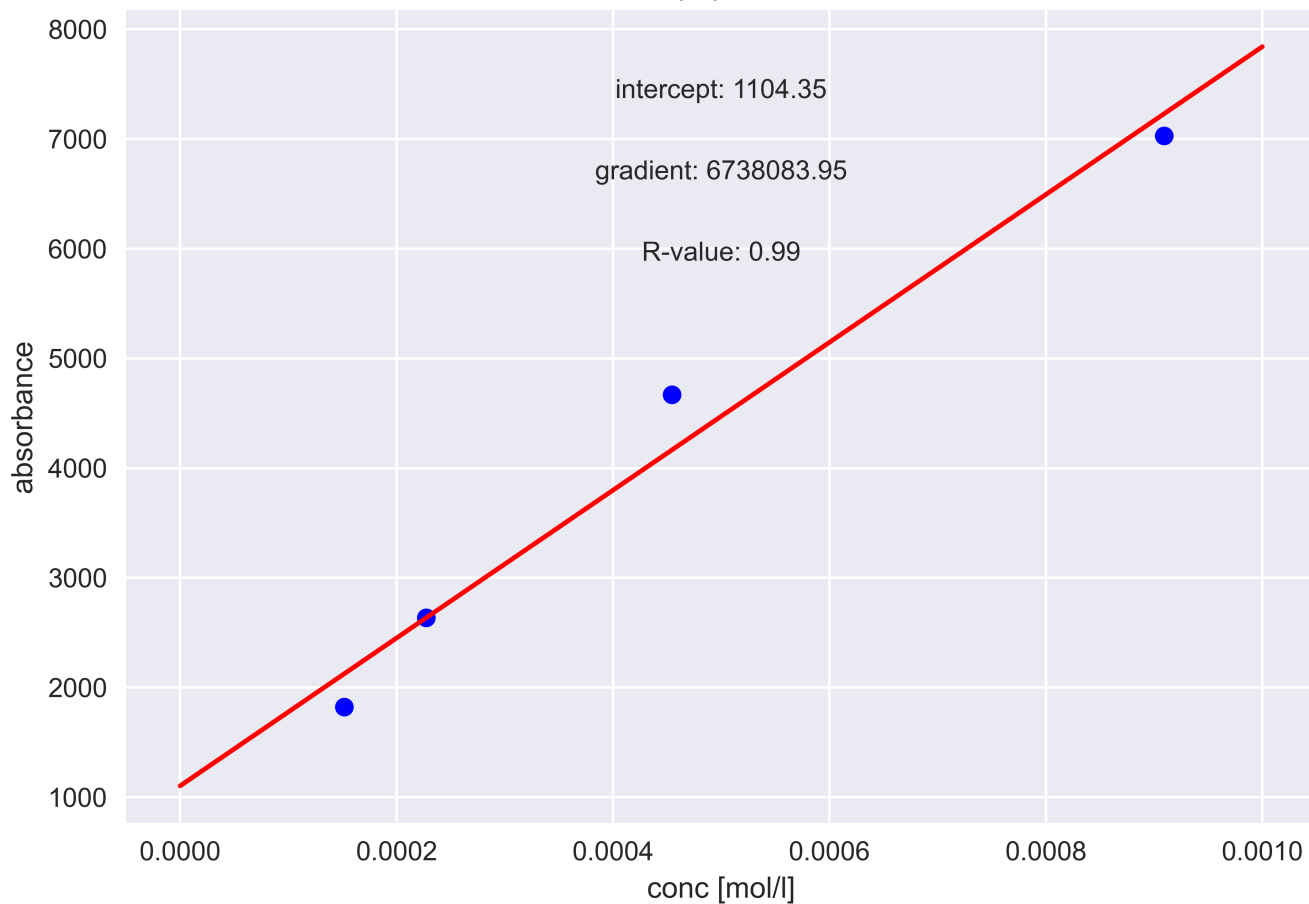

Supplement: SC-015-D3SC05607D-s003 [file SC-015-D3SC05607D-s003.pdf]

## Falcon GPBO Optimizer

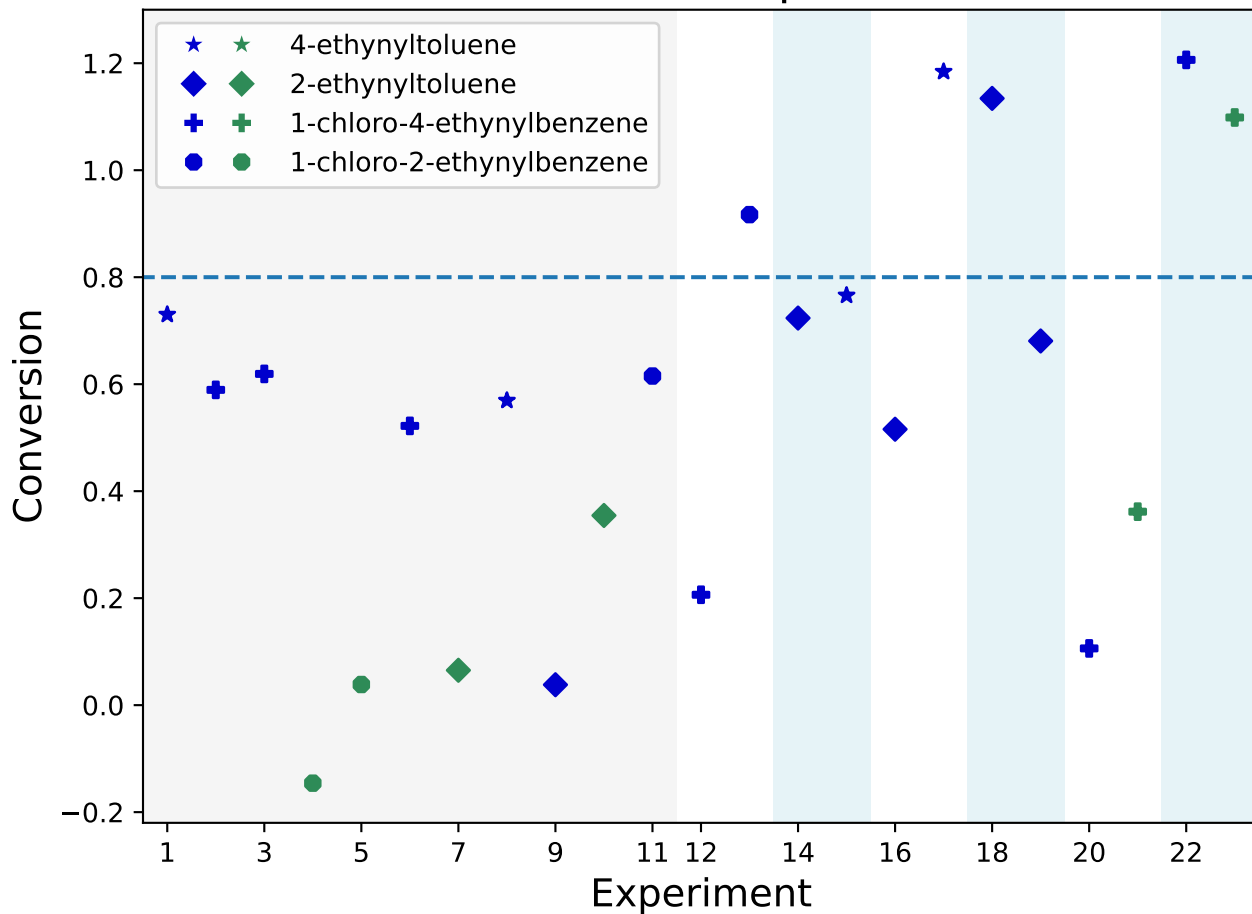

Supplement: SC-015-D3SC05607D-s004 [file SC-015-D3SC05607D-s004.pdf]

1-(iodoethynyl)-2-methylbenzene 254nm

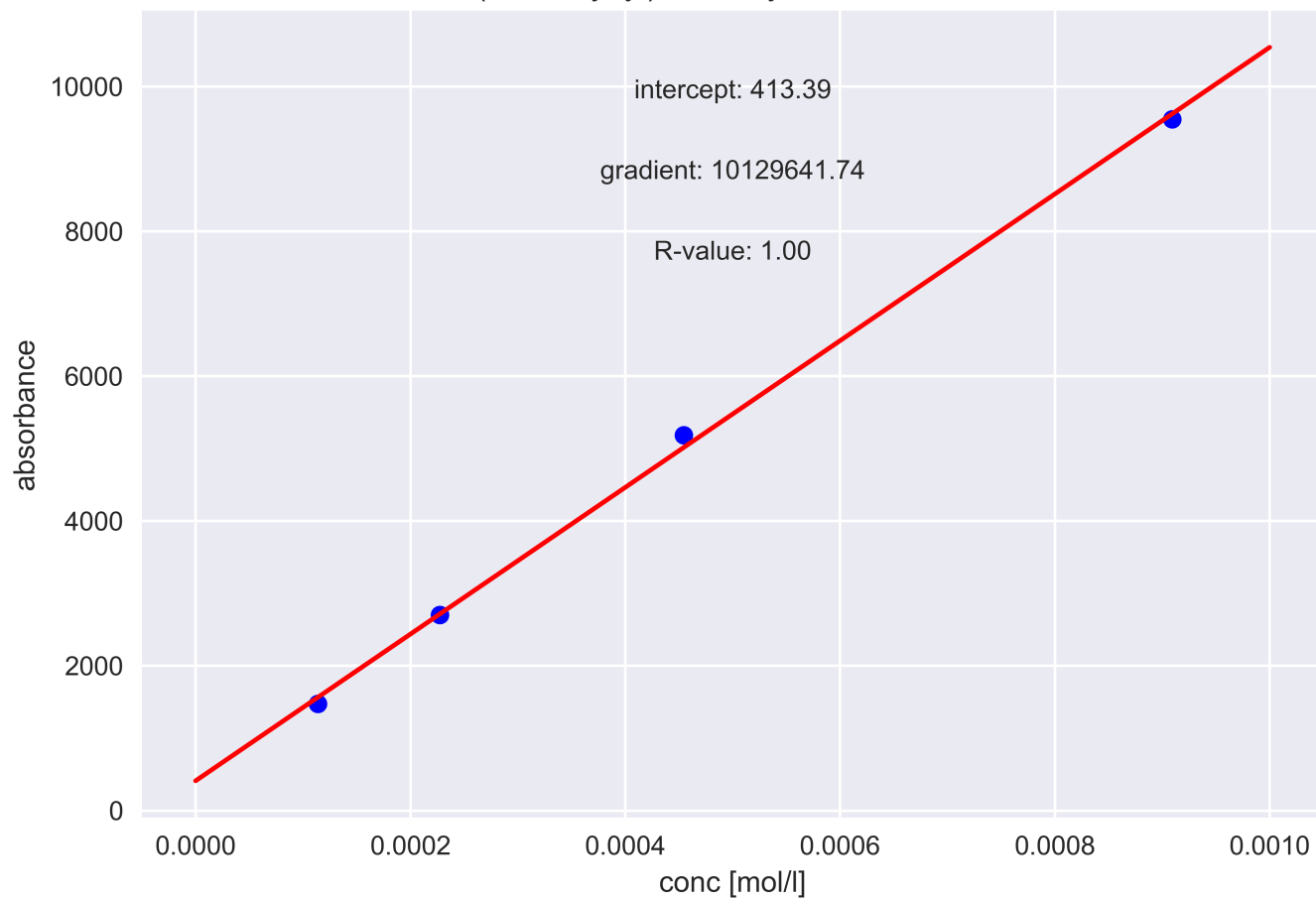

Supplement: SC-015-D3SC05607D-s005 [file SC-015-D3SC05607D-s005.pdf]

1-(iodoethynyl)-4-methylbenzene 254nm

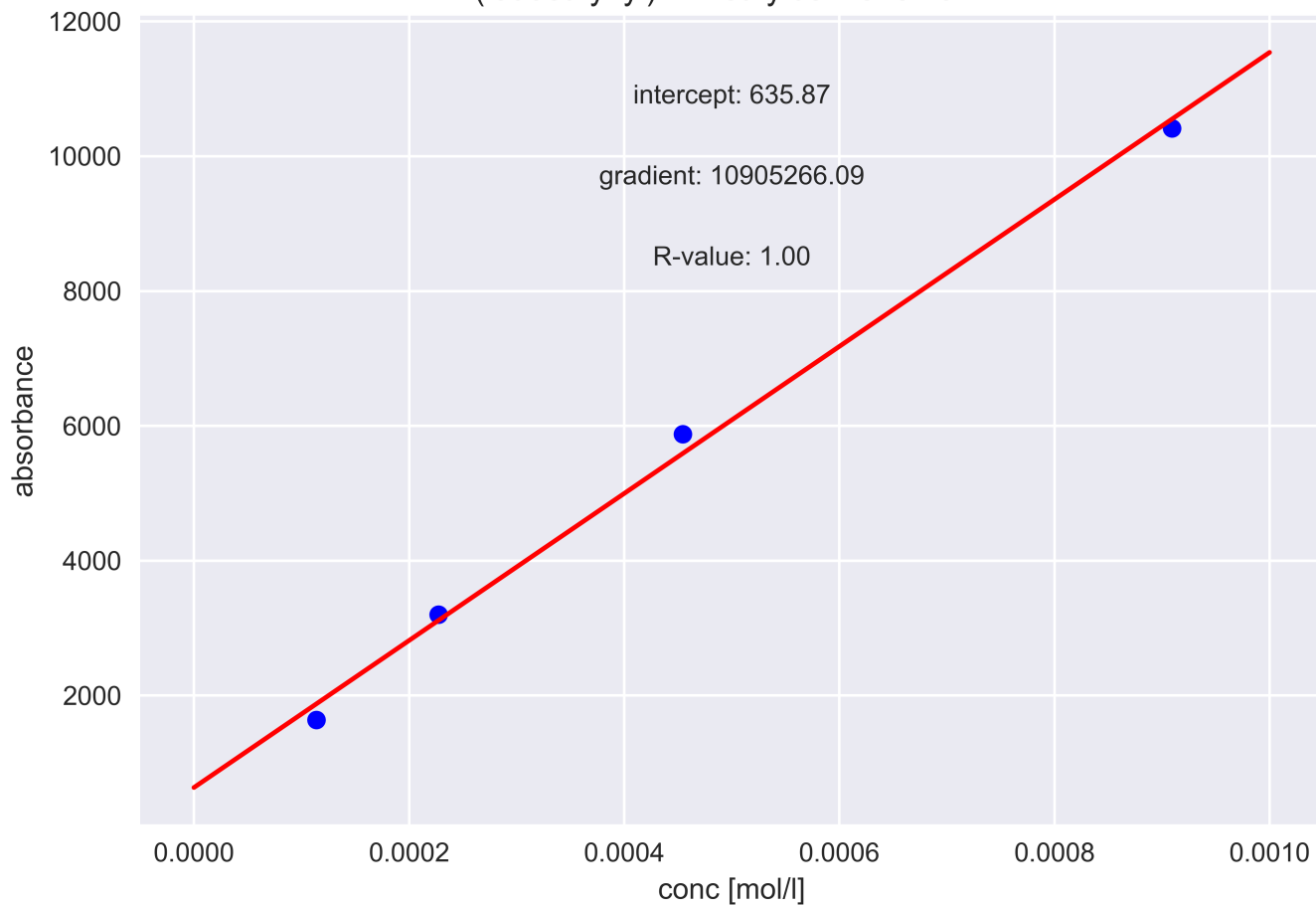

Supplement: SC-015-D3SC05607D-s006 [file SC-015-D3SC05607D-s006.pdf]

1-chloro-2-(iodoethynyl)benzene 254nm

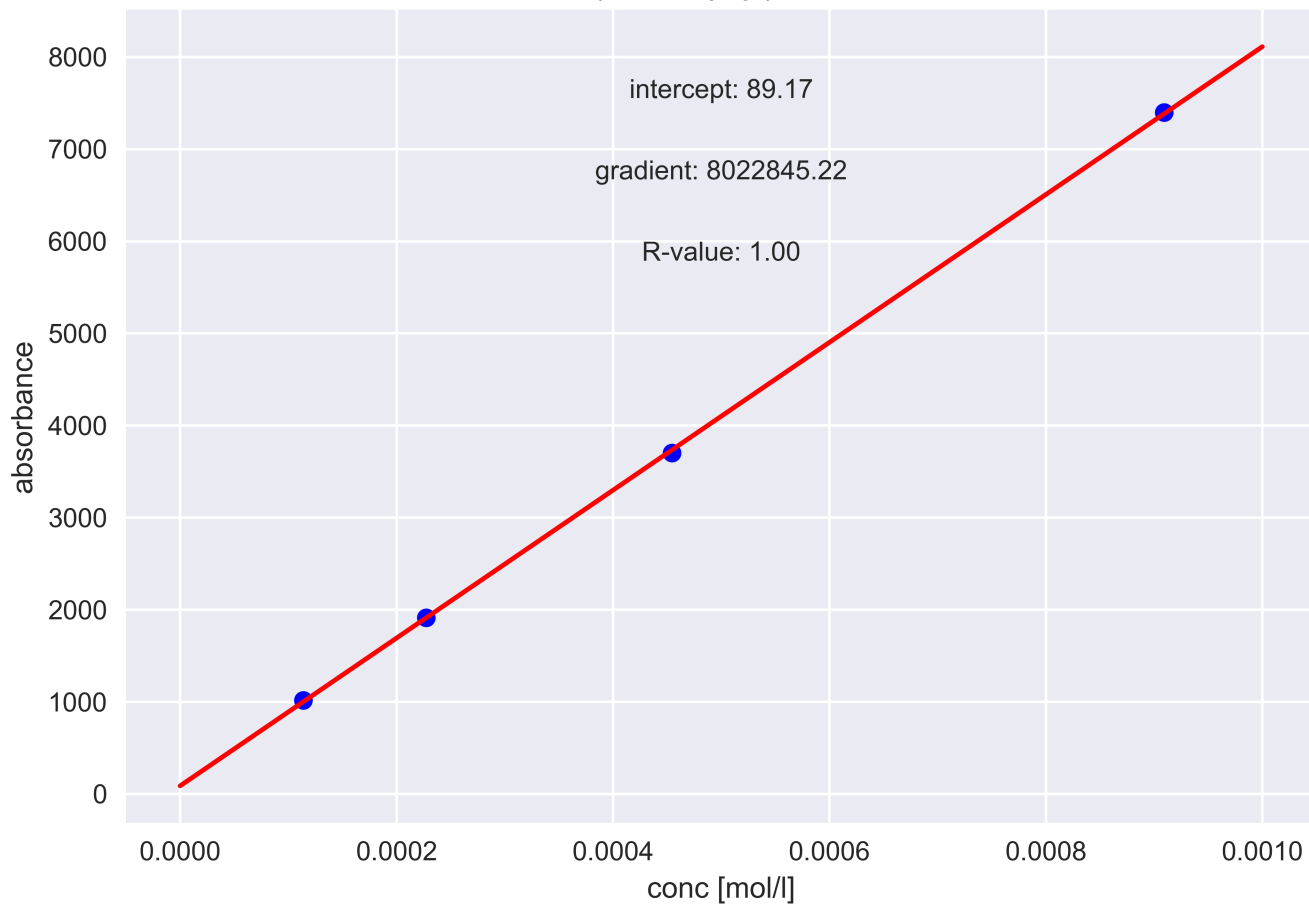

Supplement: SC-015-D3SC05607D-s007 [file SC-015-D3SC05607D-s007.pdf]

1-chloro-4-(iodoethynyl)benzene 254nm

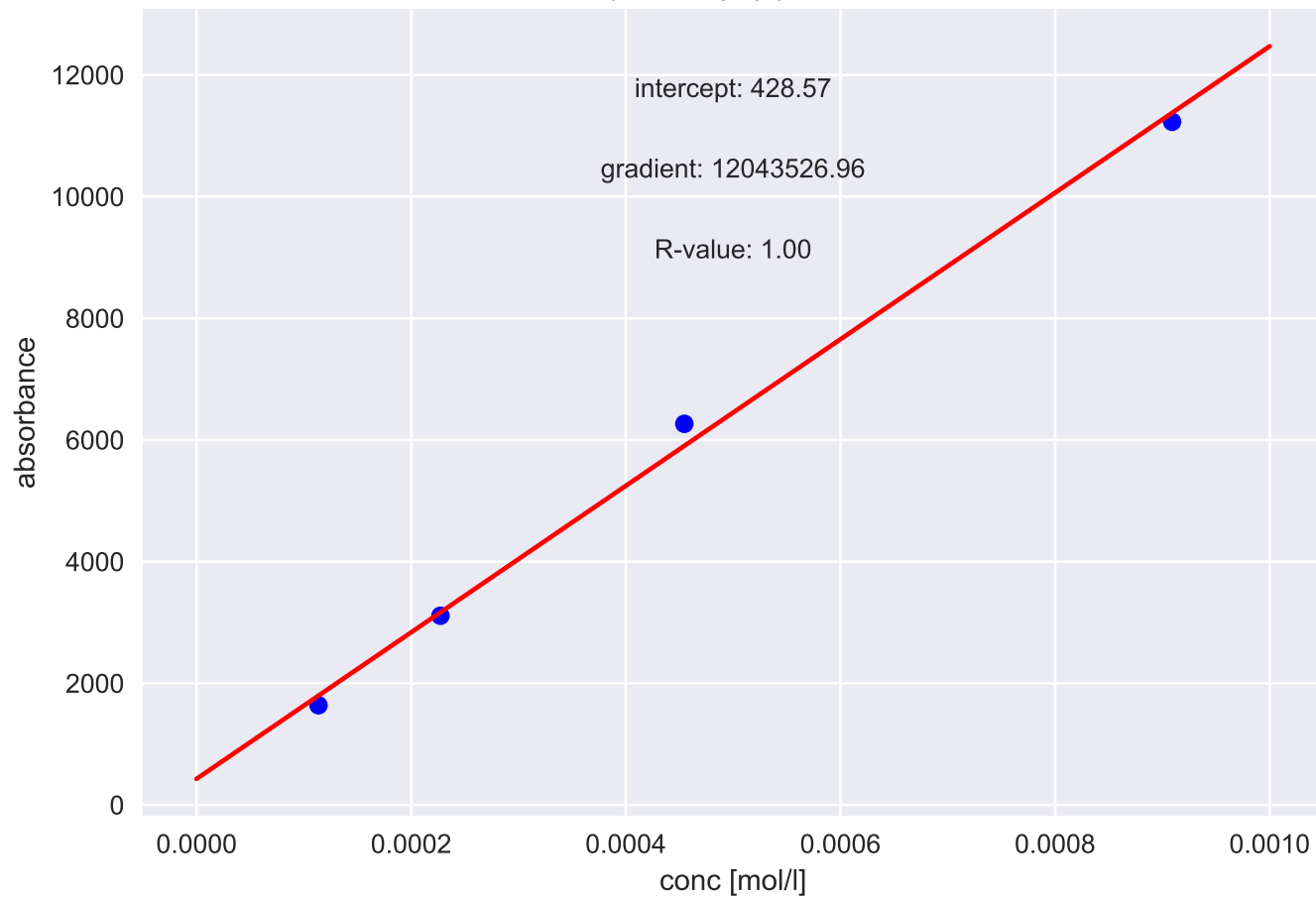

Supplement: SC-015-D3SC05607D-s008 [file SC-015-D3SC05607D-s008.pdf]

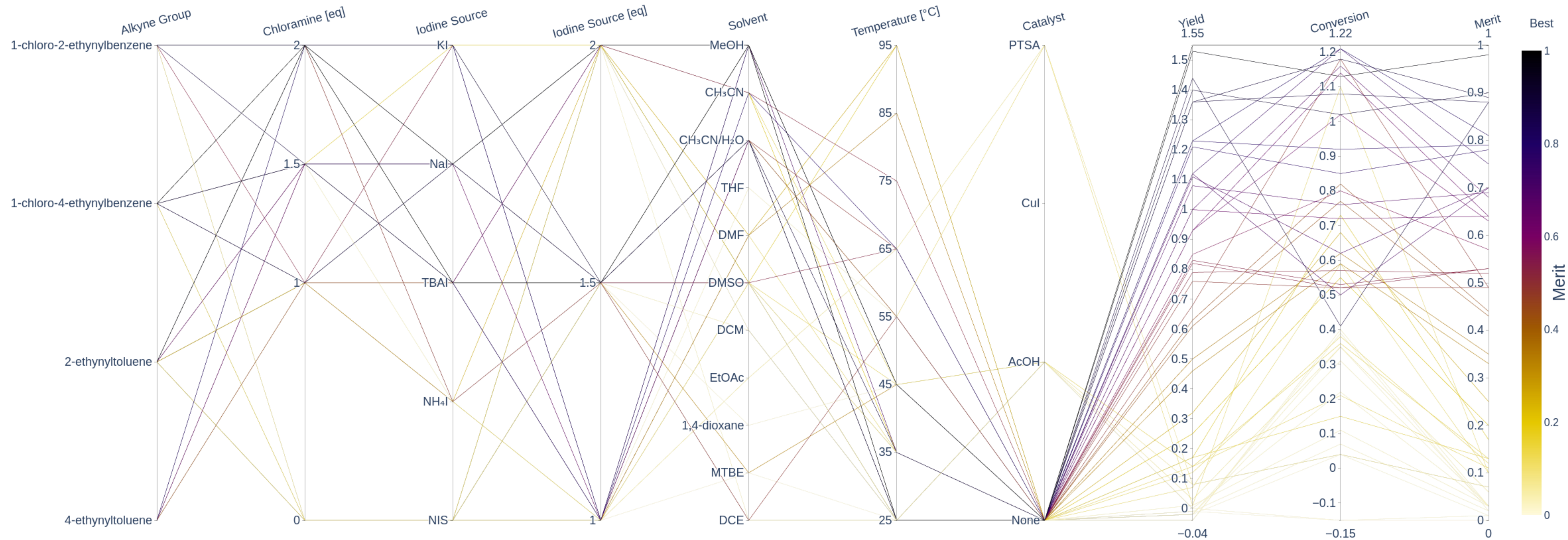

Supplement: SC-015-D3SC05607D-s009 [file SC-015-D3SC05607D-s009.pdf]

## Falcon DNGO Optimizer

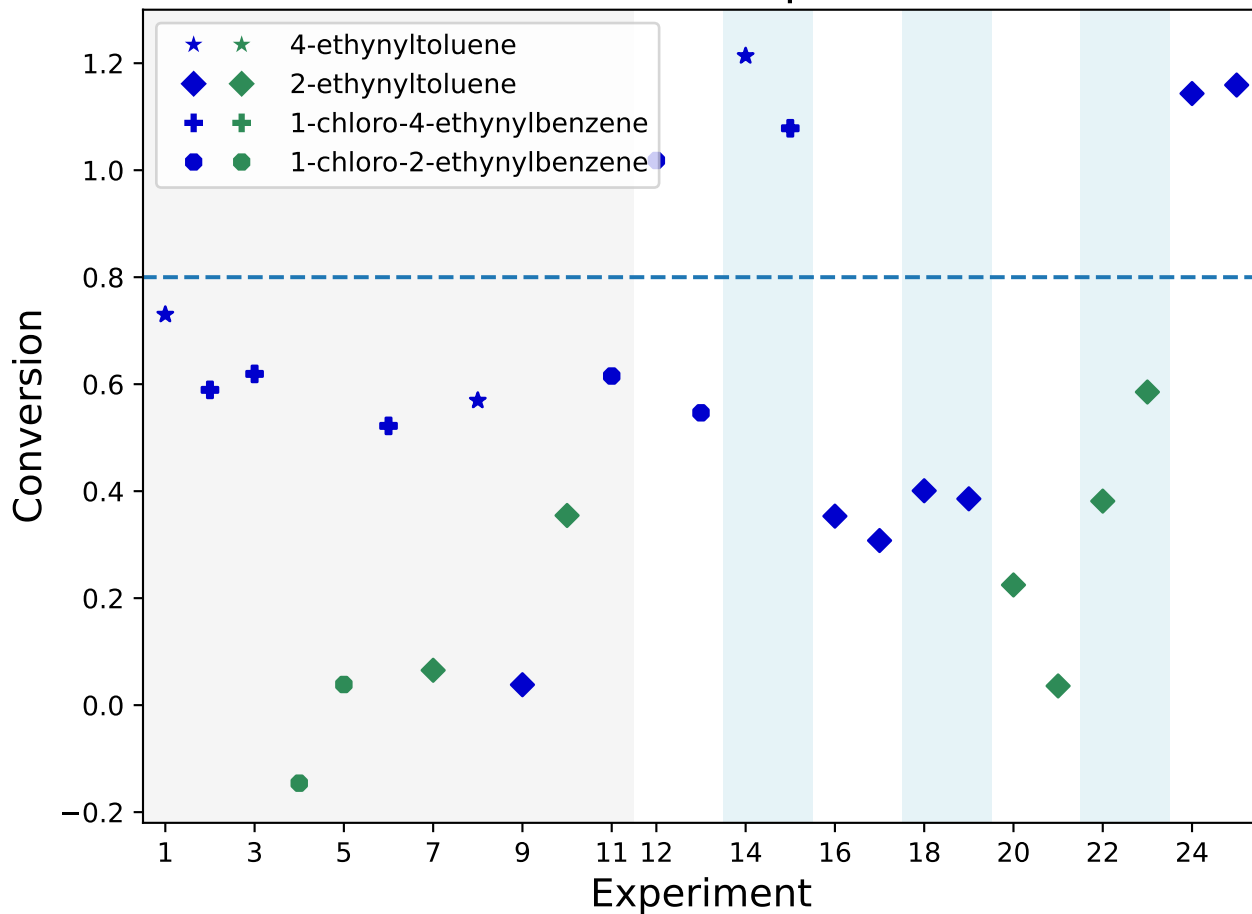

Supplement: SC-015-D3SC05607D-s010 [file SC-015-D3SC05607D-s010.pdf]

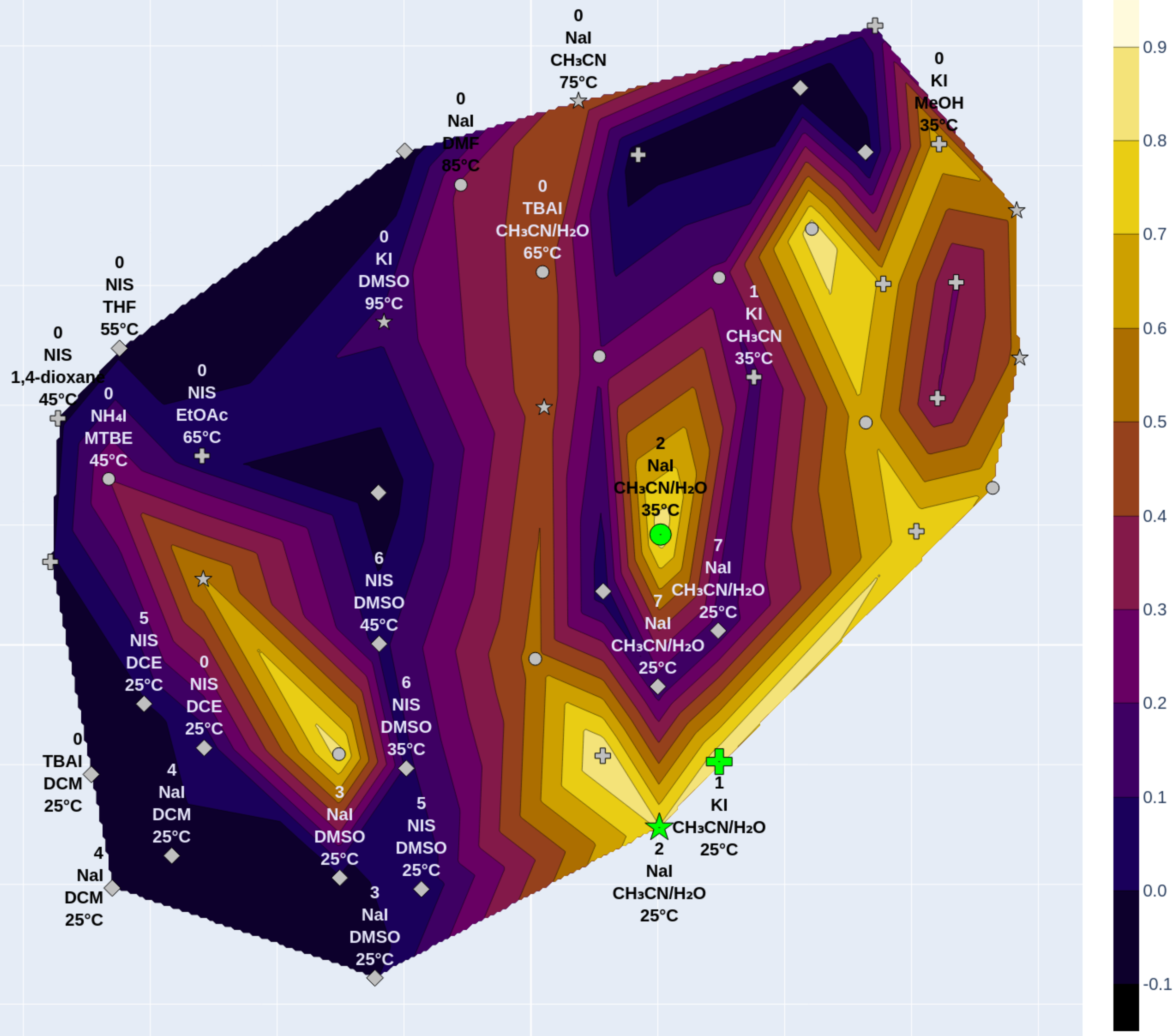

● Observation1-chloro-4-ethynylbenzene Converged

Supplement: SC-015-D3SC05607D-s011 [file SC-015-D3SC05607D-s011.pdf]

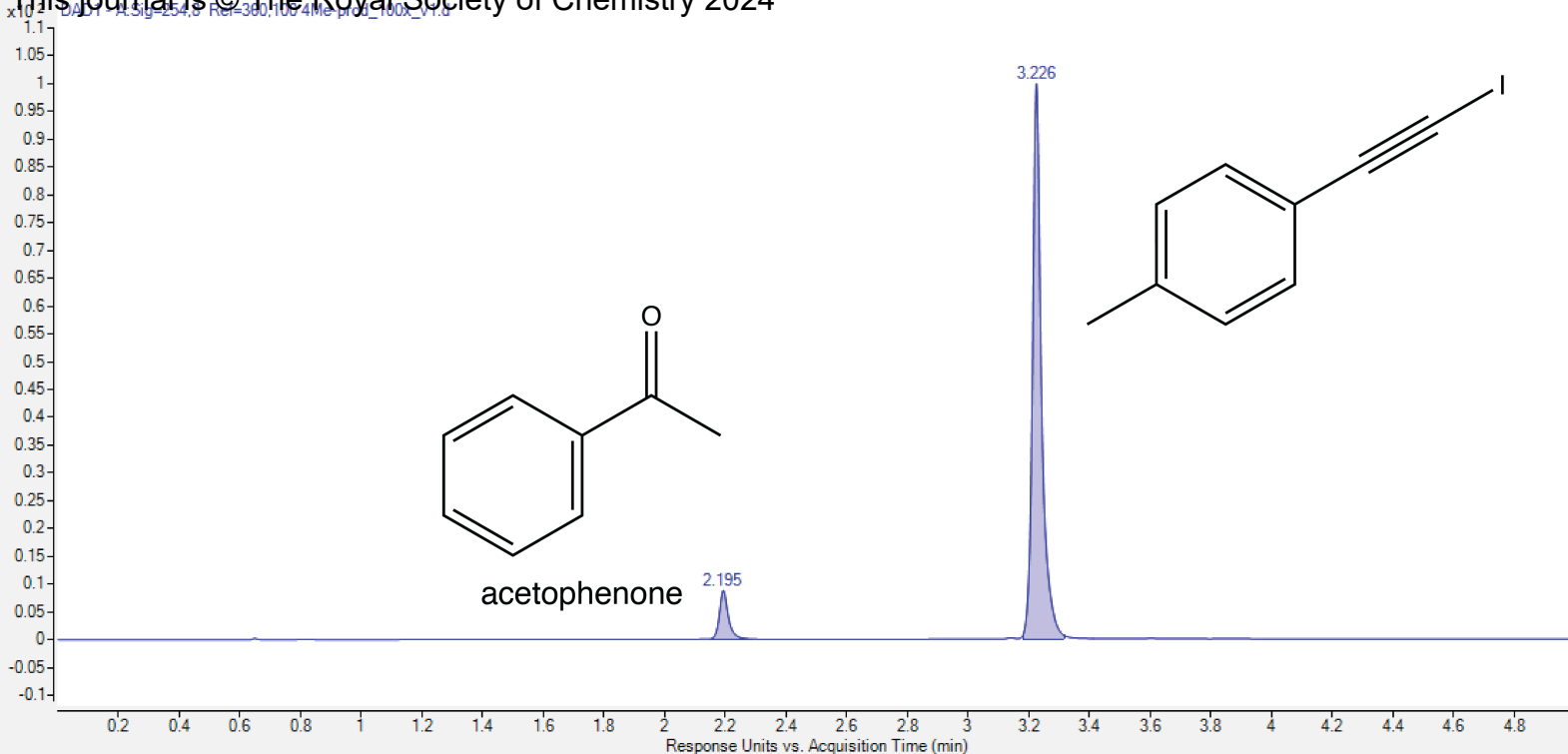

Supplement: SC-015-D3SC05607D-s012 [file SC-015-D3SC05607D-s012.pdf]

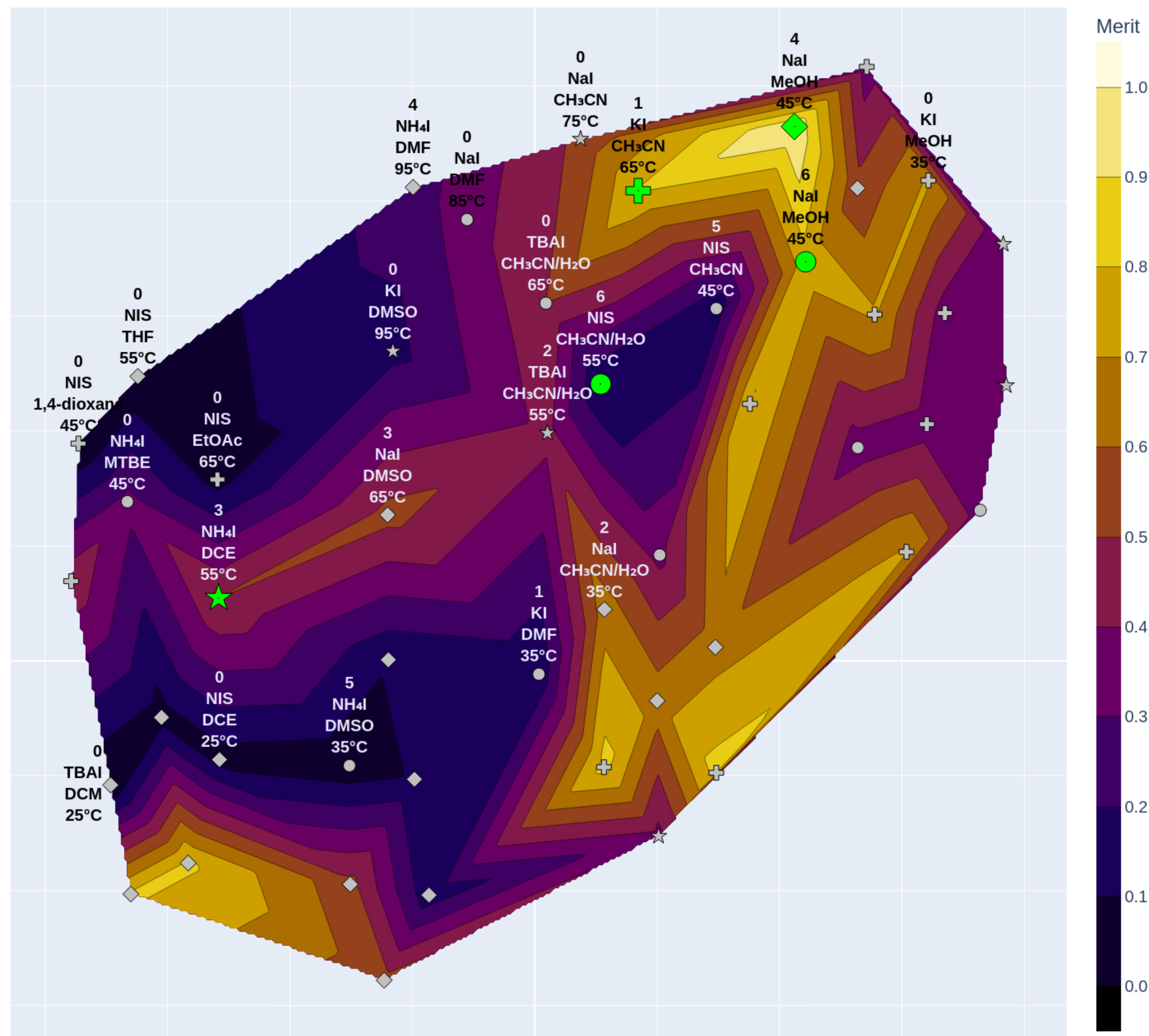

Supplement: SC-015-D3SC05607D-s013 [file SC-015-D3SC05607D-s013.pdf]

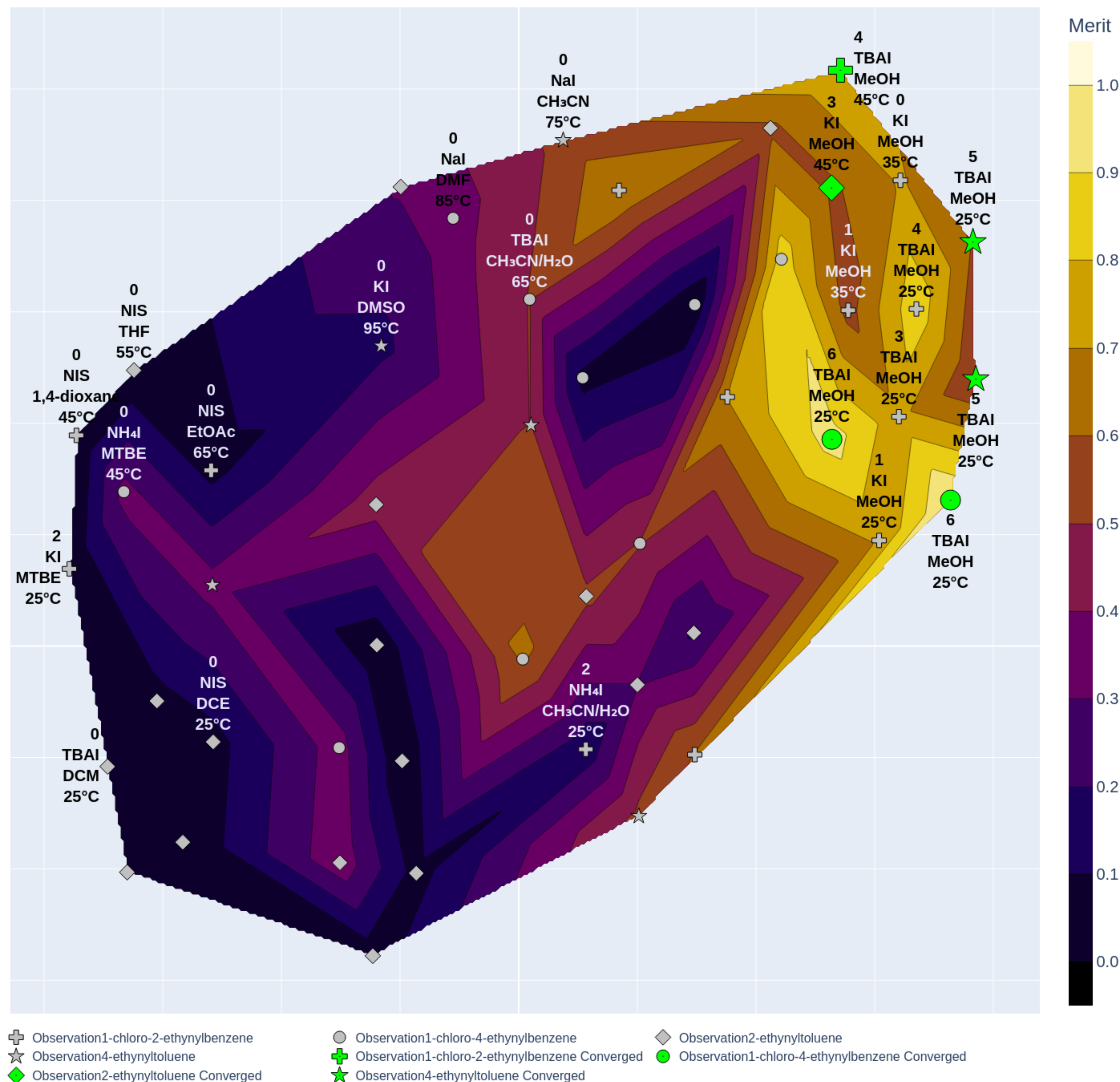

Supplement: SC-015-D3SC05607D-s014 [file SC-015-D3SC05607D-s014.pdf]

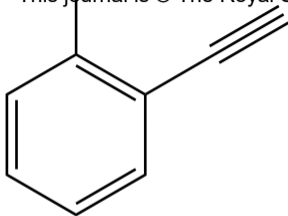

1.5 eq. NIS  
0.1 eq AcOH

18.4ml DCE  
25 °C, 2h

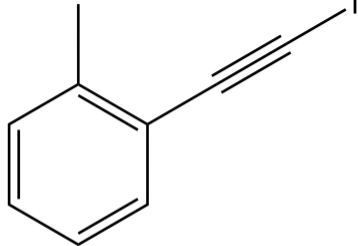

**Conversion: 35%**  
**Yield: 2.8%**

Supplement: SC-015-D3SC05607D-s015 [file SC-015-D3SC05607D-s015.pdf]

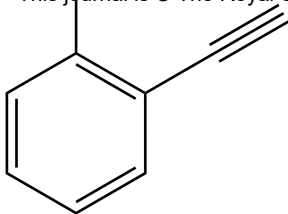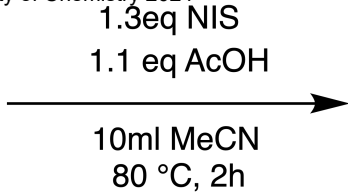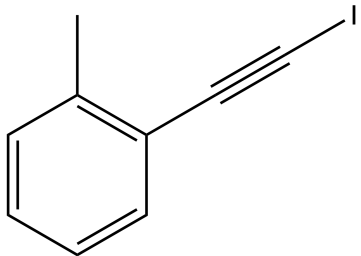

Supplement: SC-015-D3SC05607D-s016 [file SC-015-D3SC05607D-s016.pdf]

DAD1 - A:Sig=254,8 Ref=360,100 2Me-prod\_100x\_v2.d

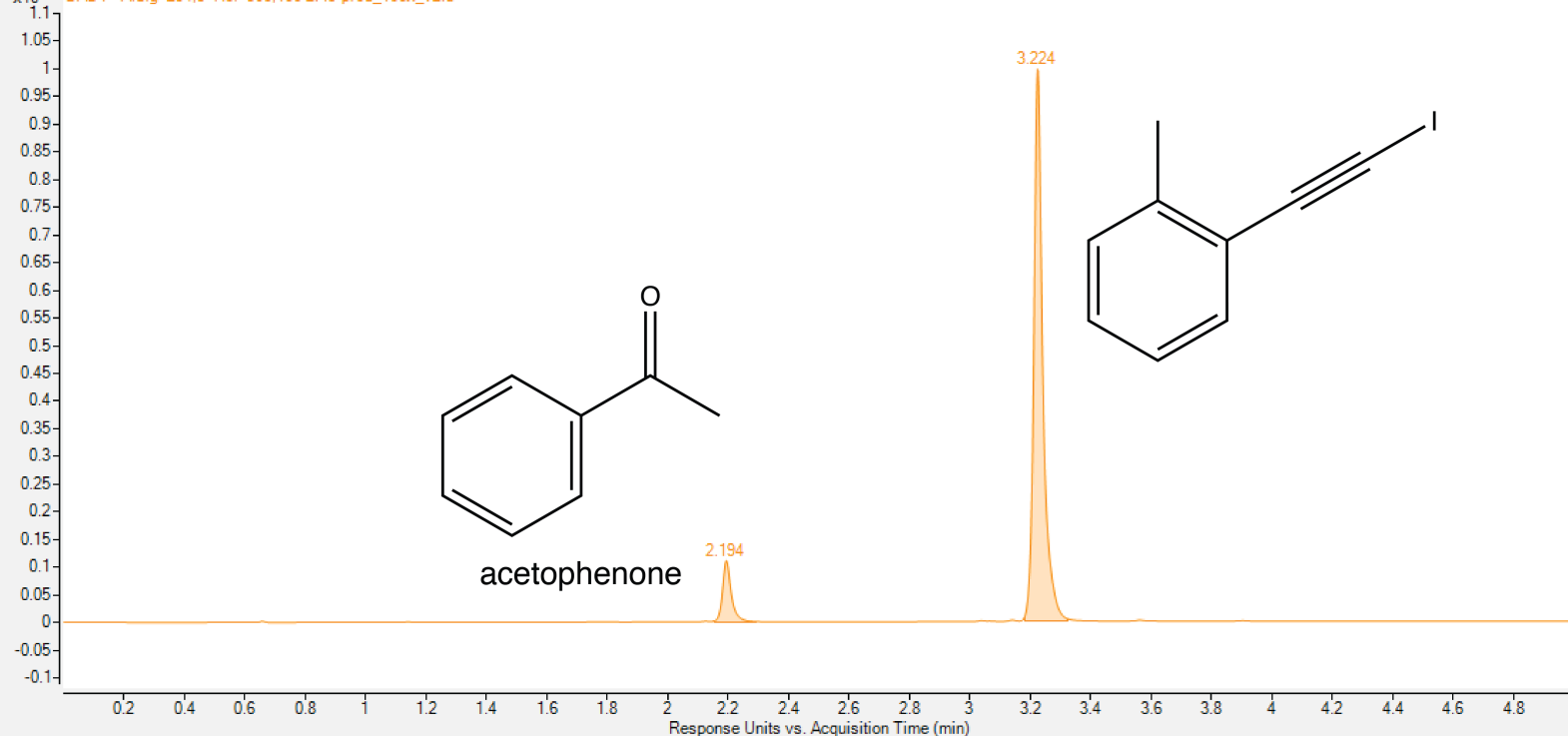

Supplement: SC-015-D3SC05607D-s017 [file SC-015-D3SC05607D-s017.pdf]

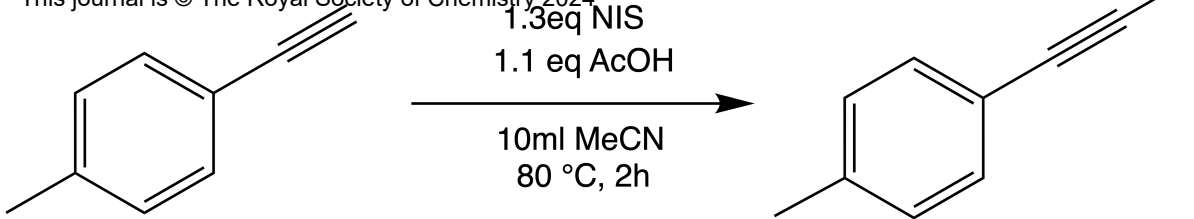

Supplement: SC-015-D3SC05607D-s018 [file SC-015-D3SC05607D-s018.pdf]

1-(iodoethynyl)-4-methylbenzene 254nm

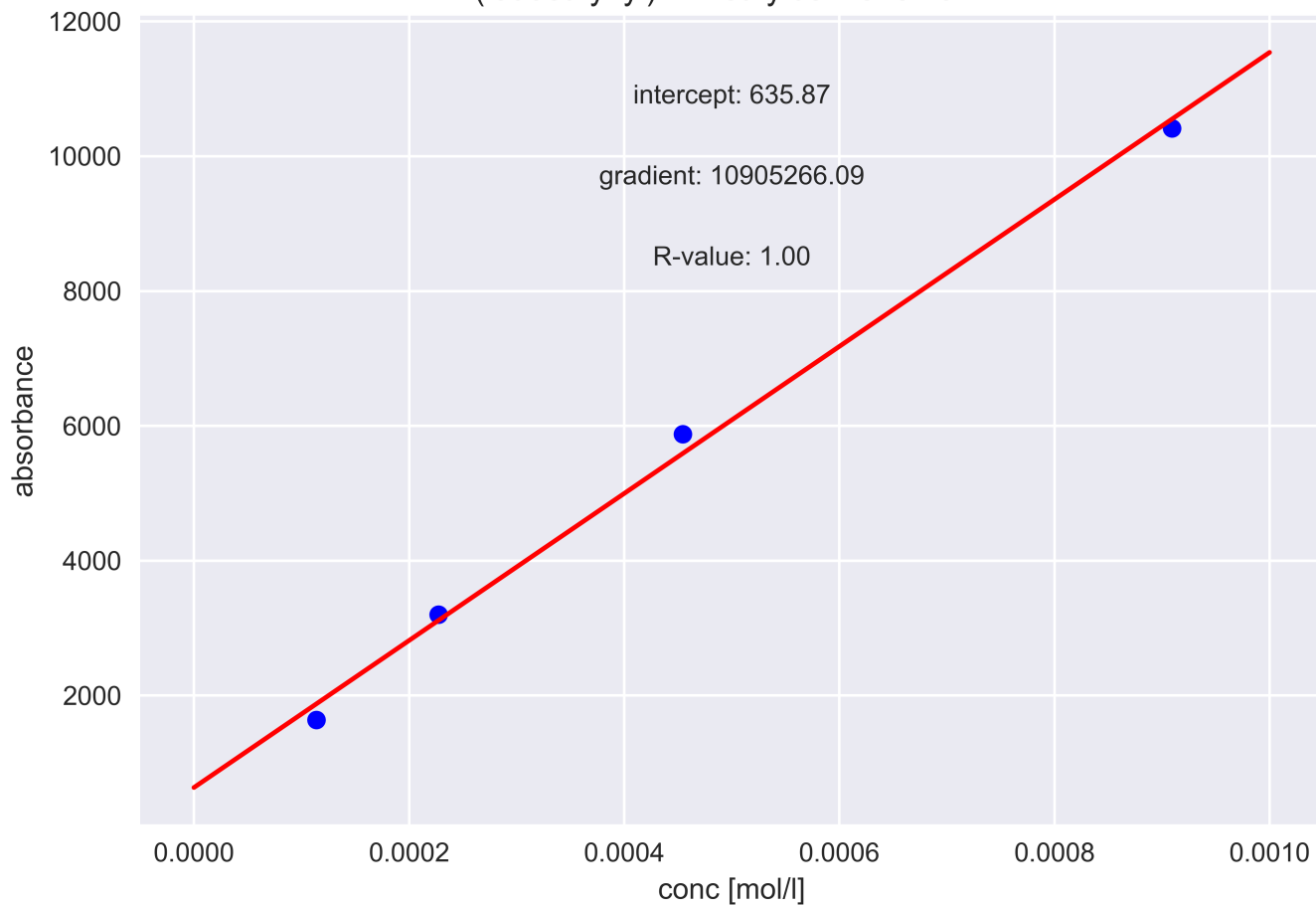

Supplement: SC-015-D3SC05607D-s019 [file SC-015-D3SC05607D-s019.pdf]

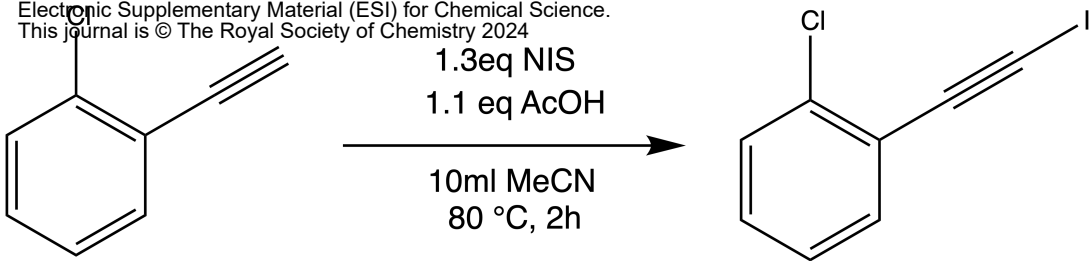

Supplement: SC-015-D3SC05607D-s020 [file SC-015-D3SC05607D-s020.pdf]

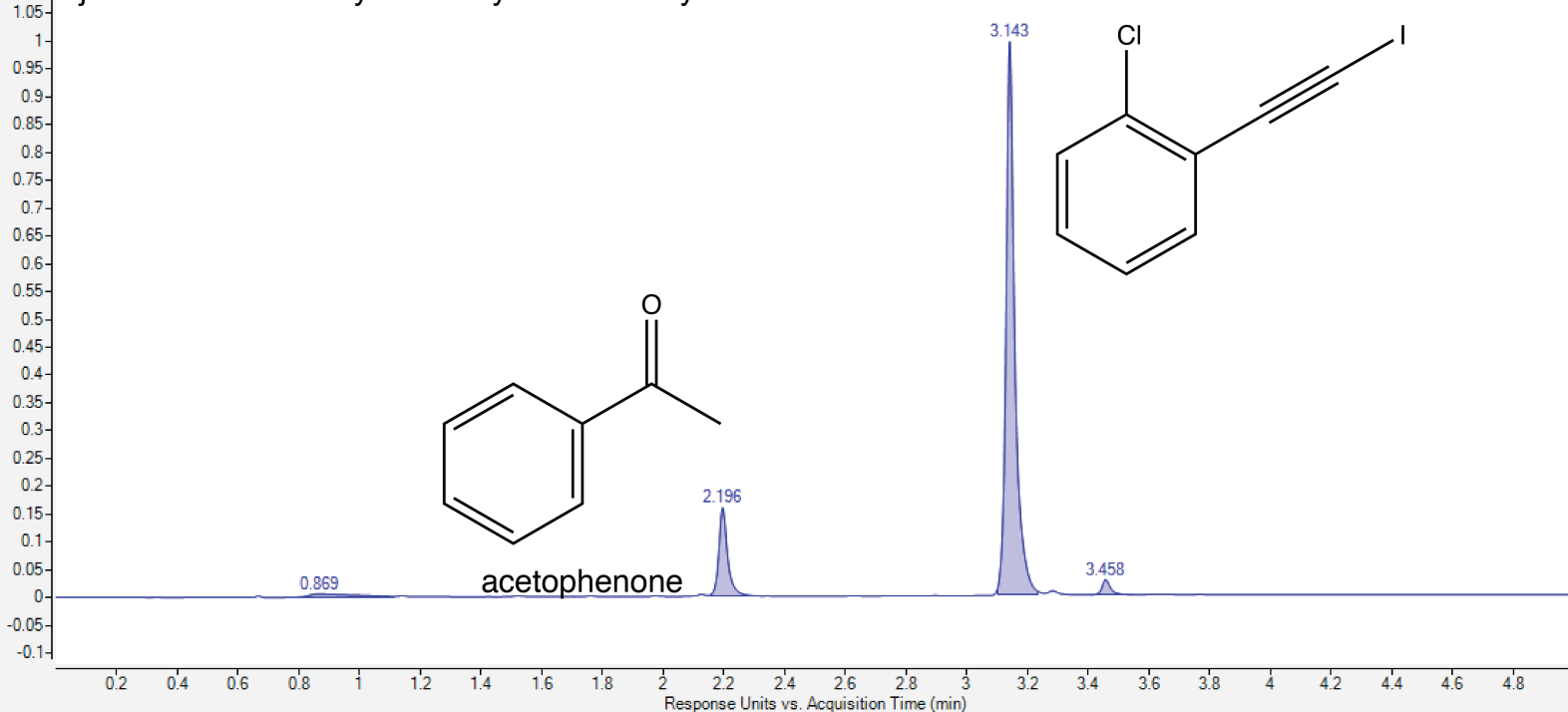

Supplement: SC-015-D3SC05607D-s021 [file SC-015-D3SC05607D-s021.pdf]

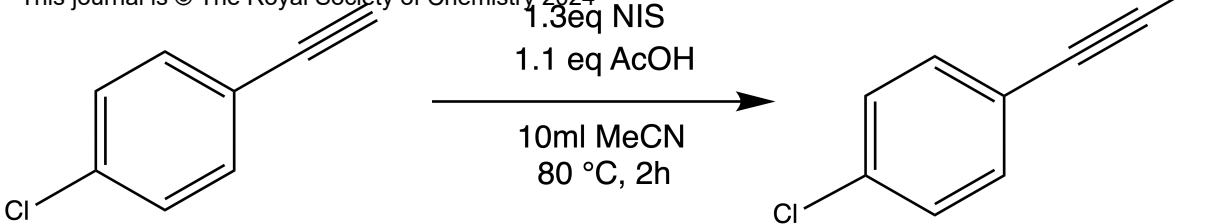

Supplement: SC-015-D3SC05607D-s022 [file SC-015-D3SC05607D-s022.pdf]

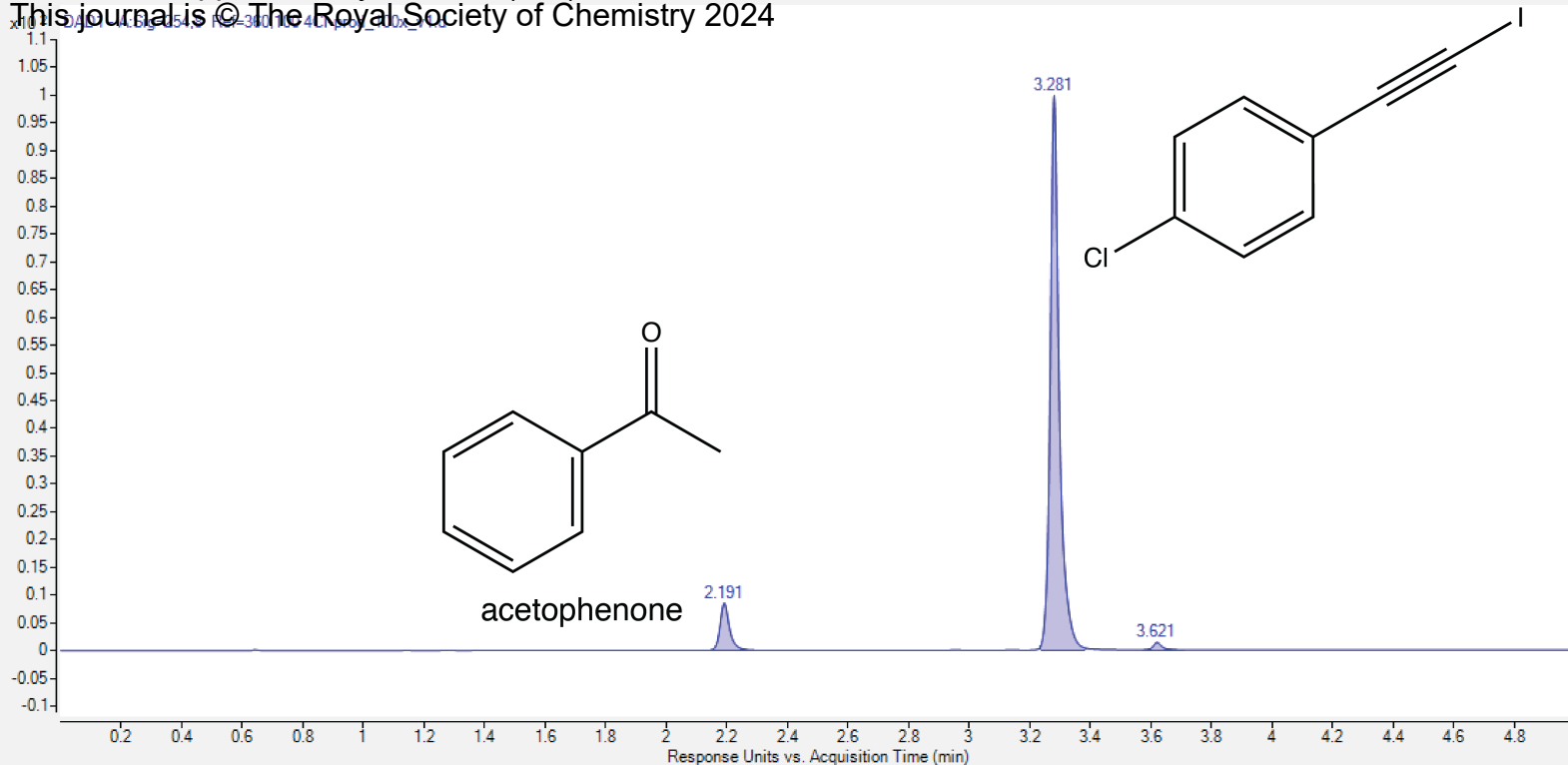

Supplement: SC-015-D3SC05607D-s023 [file SC-015-D3SC05607D-s023.pdf]

# Acetophenone 254nm

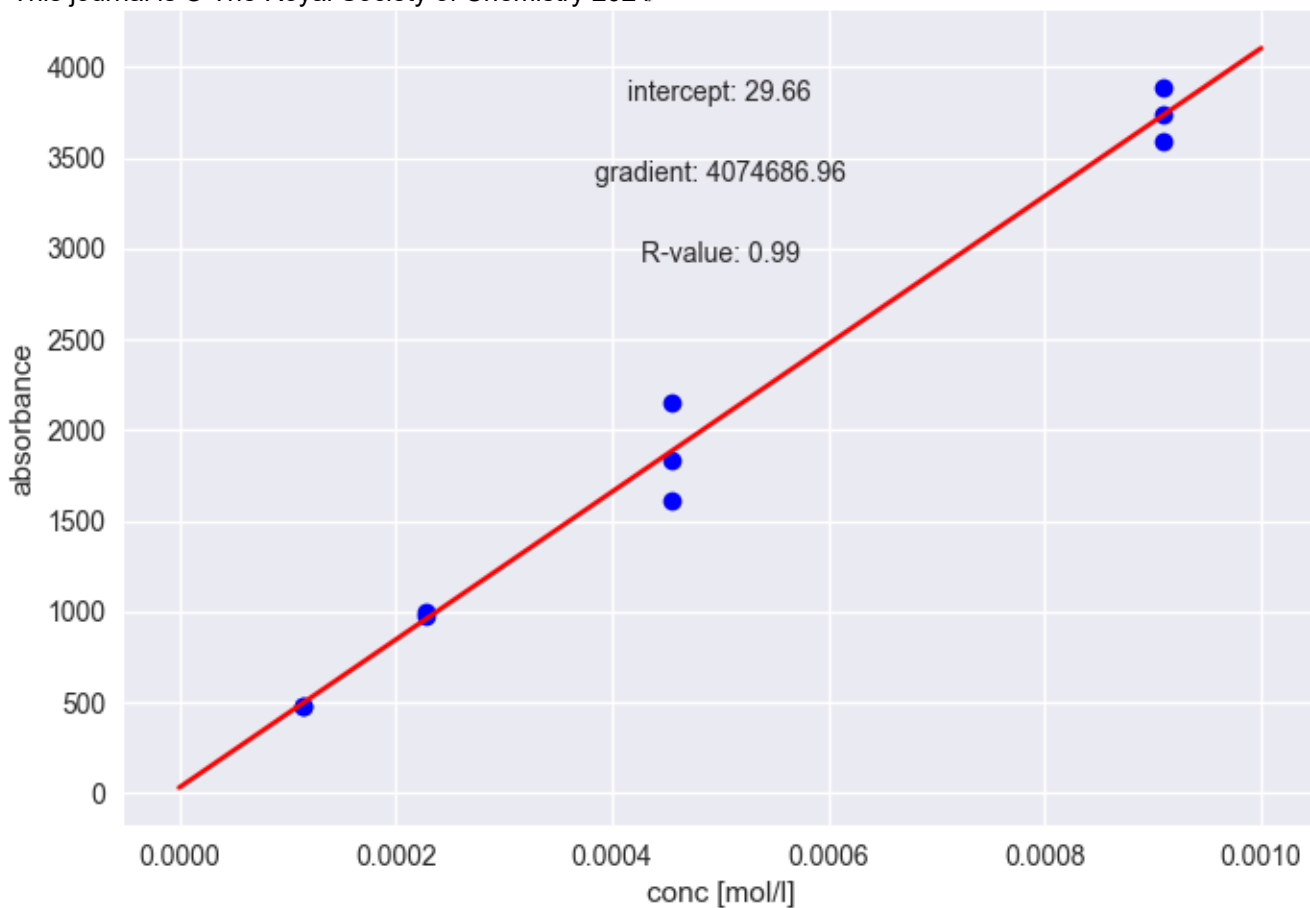

Supplement: SC-015-D3SC05607D-s024 [file SC-015-D3SC05607D-s024.pdf]

2-ethynyltoluene 254nm

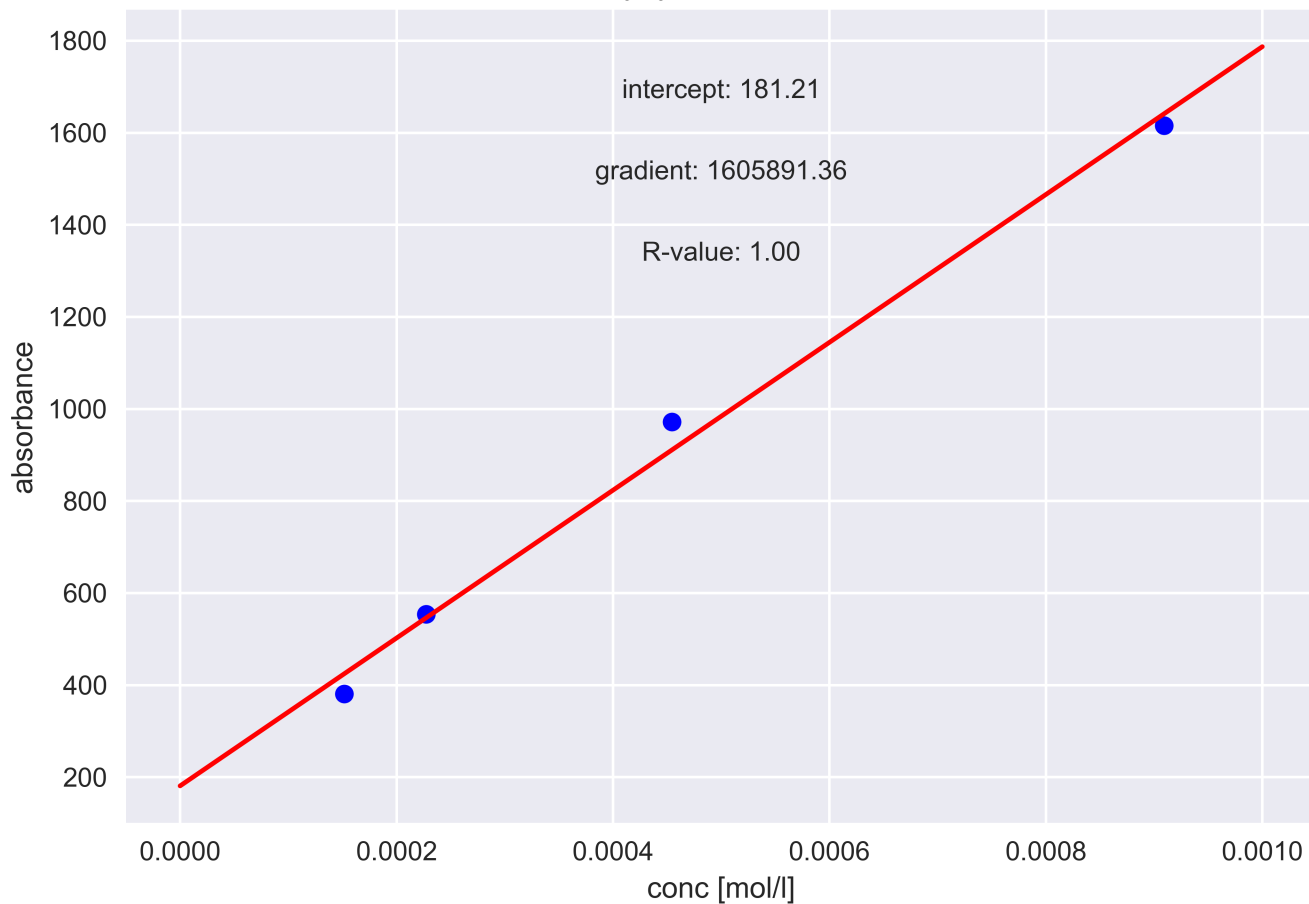

Supplement: SC-015-D3SC05607D-s025 [file SC-015-D3SC05607D-s025.pdf]

4-ethynyltoluene 254nm

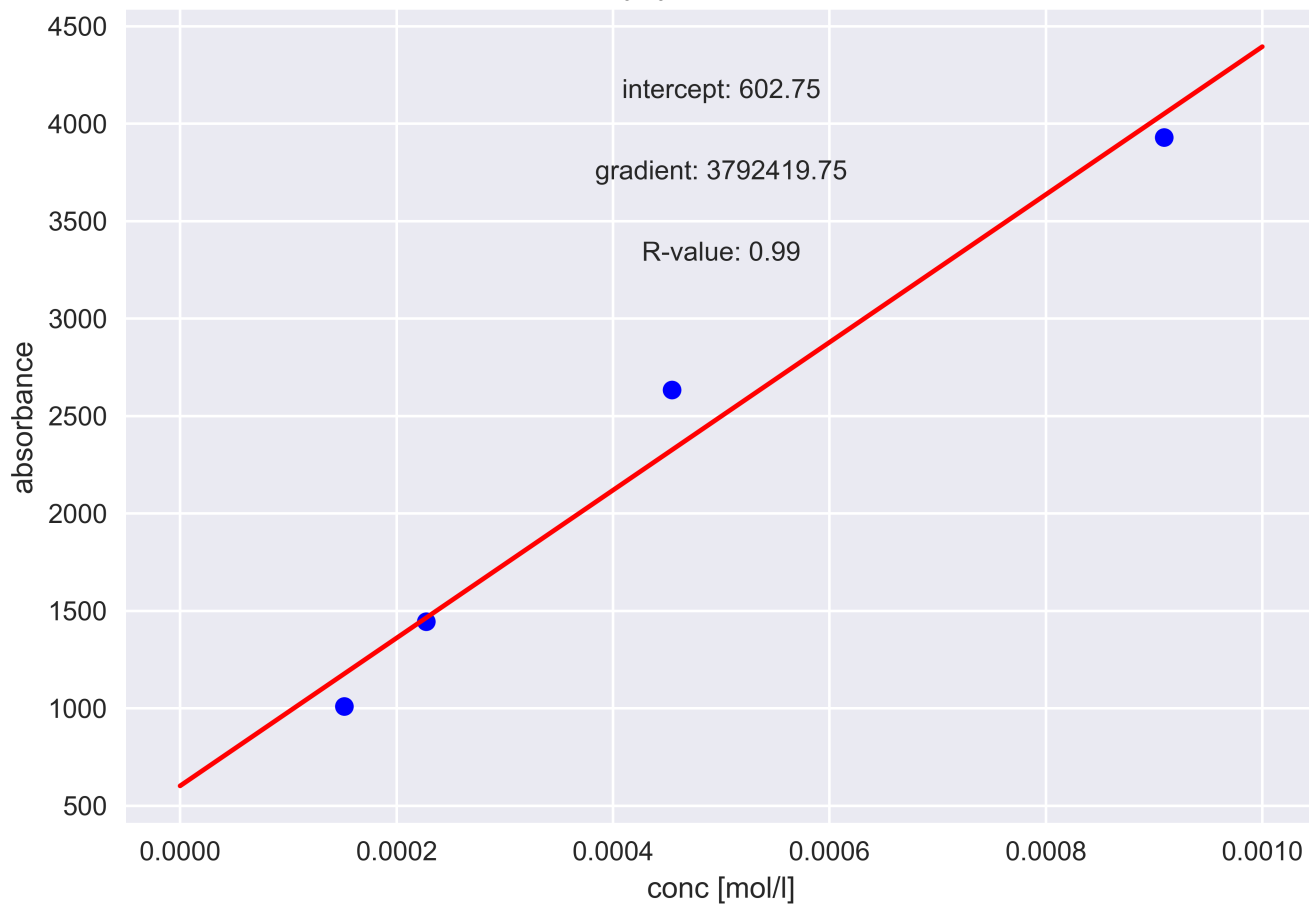

Supplement: SC-015-D3SC05607D-s026 [file SC-015-D3SC05607D-s026.pdf]
